# Nash Equilibria in Four-Strategy Quantum Extensions of the Prisoner’s Dilemma Game

**DOI:** 10.3390/e27070755

**Published:** 2025-07-15

**Authors:** Piotr Frąckiewicz, Anna Gorczyca-Goraj, Krzysztof Grzanka, Katarzyna Nowakowska, Marek Szopa

**Affiliations:** 1 Institute of Exact and Technical Sciences, Pomeranian University in Słupsk, ul. Bohaterów Westerplatte 64, 76-200 Słupsk, Poland; piotr.frackiewicz@upsl.edu.pl (P.F.); katarzyna.nowakowska@upsl.edu.pl (K.N.); 2 Department of Operations Research, University of Economics in Katowice, ul. Bogucicka 3, 40-287 Katowice, Poland; krzysztof.grzanka@uekat.pl (K.G.); marek.szopa@uekat.pl (M.S.)

**Keywords:** game isomorphism, Eisert–Wilkens–Lewenstein scheme, quantum extended games, Nash equilibrium, Prisoner’s Dilemma

## Abstract

The concept of Nash equilibria in pure strategies for quantum extensions of the general form of the Prisoner’s Dilemma game is investigated. The process of quantization involves incorporating two additional unitary strategies, which effectively expand the classical game. We consider five classes of such quantum games, which remain invariant under isomorphic transformations of the classical game. The resulting Nash equilibria are found to be more closely aligned with Pareto-optimal solutions than those of the conventional Nash equilibrium outcome of the classical game. Our results demonstrate the complexity and diversity of strategic behavior in the quantum setting, providing new insights into the dynamics of classical decision-making dilemmas. In particular, we provide a detailed characterization of strategy profiles and their corresponding Nash equilibria, thereby extending the understanding of quantum strategies’ impact on traditional game-theoretical problems.

## 1. Introduction

The main aim of quantum game theory is to establish a method for converting problems from classical game theory into a quantum mechanical context, as explored in works such as [1,2,3,4]. The features of the transformed game are then examined employing classical game theory techniques [5,6,7,8]. Alternatively, the quantum game is analyzed using principles from quantum computing [9,10,11,12]. Recent studies also demonstrate the growing practical relevance of quantum games, with experimental and theoretical work showing quantum advantages in settings such as the Magic Square game, quantum duopolies, and multiplayer strategy games [13,14,15,16,17]. In quantum game theory, akin to classical game theory, the primary issue studied is the identification of rational strategy profiles and assessing the impact of the game’s quantum extension on the ultimate outcome. In [18], the authors investigate how the dynamic behavior of the Nash equilibrium search process is affected by different unitary operators. Reference [19] demonstrates how the structure of Nash equilibria varies with different quantization approaches. Reference [20] explores the relationship between the payoffs resulting from Nash equilibria in classical and quantum games, and those arising from correlated equilibrium. In the case where players are allowed to use the full set of unitary strategies, pure-strategy Nash equilibria typically do not exist. An analysis of best responses reveals that for any given pure strategy, there exists a counter-strategy that provides the opponent with a strictly better payoff [21,22]. In this framework, the Nash equilibrium (NE) stands out as a significant solution concept [23], considered vital for assessing the rationality of a particular strategy profile. An NE is defined as a strategy profile where no individual can improve their payoff by changing their strategy alone, assuming the strategies of others remain unchanged. This concept is relevant due to its formulation for both classical and quantum games.

The Prisoner’s Dilemma (PD) represents a traditional issue in game theory, showcasing the tension between personal rational actions and group well-being [24]. Conventionally, the PD game involves two participants, each with a binary choice between cooperating and defecting. In its typical form, the PD game possesses a unique NE where both participants opt to defect, resulting in a less favorable outcome for each. Conversely, the incorporation of quantum strategies presents opportunities to modify this equilibrium framework, which may enable more advantageous results for all participants [1,25,26].

In our earlier studies [27,28], we explored the quantum extension of classical games by employing the Eisert–Wilkens–Lewenstein (EWL) framework [1], wherein we enhanced the classical strategies with additional unitary strategies. Quantum extensions have been categorized into several distinct groups, according to the features of allowable quantum strategies that maintain invariance under isomorphic transformations of the classical game. Our main objective was to pinpoint the conditions that ensure these quantum games retain the structural properties of the original, while broadening the strategic options for the players.

The main objective of this research is to conduct an in-depth examination of these extensions by identifying each NE within the pure strategy profiles of the quantum-enhanced PD. By investigating the PD in its broadest form and assessing any allowable set of payoffs, the study aims to establish the conditions required to achieve an NE for each type of extension. In particular, this work evaluates the requirements the payoff matrix must meet for a particular pure strategy profile to qualify as an NE across all identified categories of quantum extensions.

This thorough analysis offers a detailed characterization of strategic profiles and their associated NE, thus advancing the understanding of the influence of quantum strategies on conventional game-theoretical issues. The study shows that the NE derived are more aligned with Pareto-optimal solutions compared to the traditional PD. At this point, it is important to note that the extensions considered do not include completely cooperative equilibria. Pareto-optimal Nash equilibria were obtained through the quantization of classical games according to the EWL scheme [1]. However, they fail to meet the criterion of independence from isomorphic transformations of the classical game and, in our opinion, do not represent an acceptable extension of the classical game [28]. This independence is essential to ensure that the process of quantizing a classical game is unambiguous, which we consider a necessary condition for referring to it as an extension. This paper contributes to not only advancing the theoretical foundations of quantum game theory but also has potential implications for areas like quantum computing and strategic decision-making [6,29], where comprehending intricate interactive dynamics is essential.

The paper is organized into five sections. The Section 2 provides definitions of key concepts such as PD, NE, and the EWL quantum game framework. It also shows that positive affine transformations of classical game payoffs do not change the preference relations in the quantum game. In Section 3,we revisit five categories of quantum extensions for the classical 2×2 game. In these extensions, quantum players are provided with two more unitary strategies besides their original classical strategies. These extensions remain invariant under isomorphic transformations of the classical game [28]. Additionally, we illustrate the symmetry present in quantum extensions of the symmetric game. In Section 4, which is split into five sub-sections, we explore the existence of NE across successive classes of extensions. This involves analyzing each of the 16 potential strategy profiles of pure strategies. Appendix A, Appendix B, Appendix C, Appendix D and Appendix E of the paper contain the proofs for the propositions discussed in this section. For existing equilibria, we present the requisite conditions for the parameters of quantum strategies and PD payoffs that must be met.

## 2. Preliminaries

To ground the analysis in the established theory, we first present a set of definitions and propositions related to further discussion of the existence of NE. This study then aims to systematically identify all pure NE in permissible quantum extensions of the Prisoner’s Dilemma. By analyzing the game throughout a full range of payoff configurations, it establishes the conditions under which each extension admits an NE. Special focus is given to the payoff matrix requirements for a strategy profile to qualify as an equilibrium in each case.

In our research, we examine strategic form games, encompassing both traditional classical games and quantum games. A game in strategic (normal) form is formally defined as follows [30]:

**Definition** **1.** *A game in strategic form is a triple $G=(N,{\left(S_{i}\right)}_{i\in N},{\left(u_{i}\right)}_{i\in N})$ in which*

- *N={1,2,…,p} is a finite set of players;*
- *$S_{i}$ is the set of strategies of player i, for each player i∈N;*
- *$u_{i}:S_{1}\times S_{2}\times \cdots \times S_{p}\to R$ is a function that relates each vector of strategies $s={\left(s_{i}\right)}_{i\in N}$ to the payoff $u_{i}\left(s\right)$ of the player i, for each player i∈N.*

A strategic-form finite game involving two players can be represented by a bimatrix:

(1) $$\Delta =\left(\begin{array}{cccc} \left(\Delta_{11}^{1},\Delta_{11}^{2}\right) & \left(\Delta_{12}^{1},\Delta_{12}^{2}\right) & \cdots & \left(\Delta_{1m}^{1},\Delta_{1m}^{2}\right)\\ \left(\Delta_{21}^{1},\Delta_{21}^{2}\right) & \left(\Delta_{22}^{1},\Delta_{22}^{2}\right) & \cdots & \left(\Delta_{2m}^{1},\Delta_{2m}^{2}\right)\\ \vdots & \vdots & \ddots & \vdots \\ \left(\Delta_{n1}^{1},\Delta_{n1}^{2}\right) & \left(\Delta_{n2}^{1},\Delta_{n2}^{2}\right) & \cdots & \left(\Delta_{nm}^{1},\Delta_{nm}^{2}\right) \end{array}\right)=\left(\Delta^{1},\Delta^{2}\right).$$

The interpretation of such a notation is that player 1 (the row player) chooses row $i\in S_{1}$ from his set of strategies $S_{1}=\left\{1,\ldots ,n\right\}$, and player 2 (the column player) chooses column $j\in S_{2}$ from her set $S_{2}=\left\{1,\ldots ,m\right\}$. The combination of player 1 using strategy *i* and player 2 using strategy *j* will be represented as the ordered pair (i,j) and referred to as a strategy profile. As the result of the game, player 1 receives payoff $u_{1}\left(i,j\right)=\Delta_{ij}^{1}$ and player 2 receives $u_{2}\left(i,j\right)=\Delta_{ij}^{2}$. Taking into account the elements that define a game in strategic form, we can identify the payoff function of (1) as matrices $\Delta^{1}=\left(\Delta_{ij}^{1}\right)$ and $\Delta^{2}=\left(\Delta_{ij}^{2}\right)$ and denote the game (1) as $\left(\Delta^{1},\Delta^{2}\right)$.

Among the games represented by (1), we can distinguish those that have certain special characteristics. Symmetric games serve as an example of these [31].

**Definition** **2.** *Let $G=(N,\left(S_{1},S_{2}\right),\left(u_{1},u_{2}\right))$ be a two-player finite strategic game. G is said to be symmetric if $S_{1}=S_{2}$ and $u_{1}\left(s_{1},s_{2}\right)=u_{2}\left(s_{2},s_{1}\right)$ for all $s_{1}\in S_{1},s_{2}\in S_{2}$.*

In matrix notation, the fact that a game $\left(\Delta^{1},\Delta^{2}\right)$ is symmetric means that $\Delta^{2}={\left(\Delta^{1}\right)}^{T}$. One of the best-known symmetric games is the PD. It is a two-player game that can be represented by a 2×2 bimatrix in the form of

(2) $$(\begin{array}{cc} \left(R,R\right) & \left(S,T\right)\\ \left(T,S\right) & \left(P,P\right) \end{array}),\;\mathrm{where}\;T>R>P>S\;\mathrm{and}\;2R>T+S.$$

In the field of game theory, the notion of NE plays a key role as a fundamental solution concept [32]. This equilibrium represents a strategy profile such that no player can gain a better payoff by deviating from her equilibrium strategy, provided that the other players’ strategies remain unchanged. NE provides players with a certain level of stability within a game as in an NE, each player’s strategy is a best response to the strategies of the other players.

The literature offers numerous approaches to define NE based on the specific game type under consideration [30,33]. Herein, we articulate NE as they pertain to the games studied in this research, with particular emphasis on pure NE in bimatrix games.

**Definition** **3.** A strategy profile $\left(i^{*},j^{*}\right)$ is a (pure) NE if $\Delta_{i^{*}j^{*}}^{1}\geq \Delta_{ij^{*}}^{1}$ for every $i\in S_{1}$ and $\Delta_{i^{*}j^{*}}^{2}\geq \Delta_{i^{*}j}^{2}$ for every $j\in S_{2}$.

**Figure 1:**
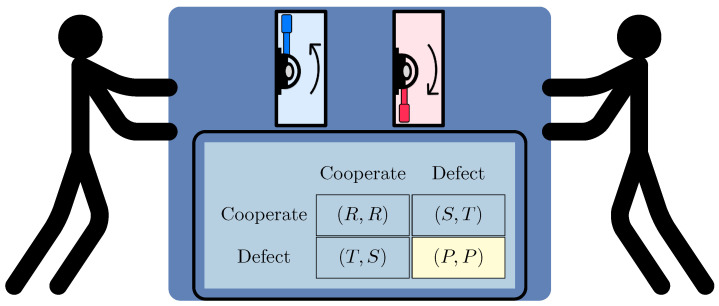
Prisoner dilemma as a bimatrix of payoffs. There is a unique Nash equilibrium in which both players defect indicated in yellow.

As an illustration, it can be readily confirmed that the unique NE in (2) is the strategy profile (2,2), which results in each player receiving a payoff of *P* (Figure 1). This equilibrium of the classical game is not Pareto-optimal, and this leads to a series of suboptimal decisions in human interactions [34]. The lack of Pareto optimality of the NE of the PD illustrates the conflict between maximizing individual payoffs (a rational strategy in the sense of game theory) and maximizing aggregate welfare (a Pareto-optimal solution). This phenomenon can be seen in many everyday and economic problems, such as pollution, the use of common resources, or lack of cooperation in business without proper contract enforcement mechanisms [35].

From Definition 2, it can be deduced that there is a symmetry of the set of NE of a two-player symmetric game: if $\left(s_{1},s_{2}\right)$ is an NE, then $\left(s_{2},s_{1}\right)$ is also an NE. Now, we review the Eisert–Wilkens–Lewenstein scheme for 2×2 bimatrix games [2].

**Definition** **4.** *The Eisert–Wilkens–Lewenstein quantization of the game given by (1) for $S_{1}=S_{2}=\left\{1,2\right\}$ is defined by the triple $\left(\left\{1,2\right\},\left\{T_{1},T_{2}\right\},\left\{v_{1},v_{2}\right\}\right)$, where*

- *{1,2} is the set of players.*
- *$T_{i}$ is a set of unitary operators from SU(2), each of the following form:*
(3) $$U_{i}\left(\theta_{i},\alpha_{i},\beta_{i}\right)=\left(\begin{array}{cc} e^{i\alpha_{i}}\cos\frac{\theta_{i}}{2} & ie^{i\beta_{i}}\sin\frac{\theta_{i}}{2}\\ ie^{-i\beta_{i}}\sin\frac{\theta_{i}}{2} & e^{-i\alpha_{i}}\cos\frac{\theta_{i}}{2} \end{array}\right),\;\theta_{i}\in \left[0,\pi \right],\;\alpha_{i},\beta_{i}\in \left[0,2\pi \right).$$
*Each player i, by choosing $U_{i}\in T_{i}$, determines the final quantum state |ψ〉 as*
(4) $$|\psi \rangle =J^{†}\left(U_{1}\left(\theta_{1},\alpha_{1},\beta_{1}\right)⊗U_{2}\left(\theta_{2},\alpha_{2},\beta_{2}\right)\right)J|00\rangle ,$$
  *where $J=\frac{1}{\sqrt{2}}\left(I⊗I+i\sigma_{x}⊗\sigma_{x}\right)$ is the entangling operator.*
- *$v_{i}:T_{1}\times T_{2}\to R$ is the payoff function for player i. It is defined as the expected value of the measurement operator $M_{i}$, where*
(5) $$M_{i}=\sum_{k,l\in \{1,2\}}\Delta_{kl}^{i}|k-1,l-1\rangle \langle k-1,l-1|,$$
  *and $\Delta_{kl}^{i}$ are the payoffs from the classical 2×2 bimatrix game (1). The function $v_{i}$ is given by*
(6) $$\begin{array}{c} v_{i}\left(U_{1},U_{2}\right)=\mathrm{tr}\left(\left|\psi \rangle \langle \psi \right|M_{i}\right). \end{array}$$

**Figure 2:**
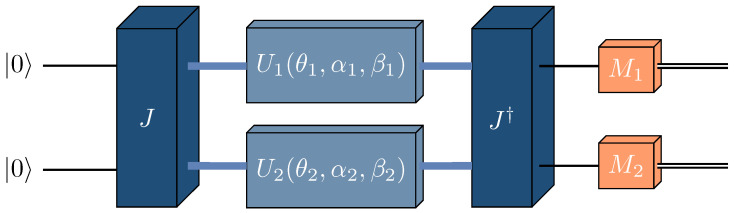
The EWL scheme.

The EWL quantization scheme is represented schematically in Figure 2.

Using Formula (6) we can determine the explicit form of the pair of players’ payoffs,

(7) $$\begin{array}{cc} \; & \left(u_{1},u_{2}\right)\left(U_{1}\left(\theta_{1},\alpha_{1},\beta_{1}\right),U_{2}\left(\theta_{2},\alpha_{2},\beta_{2}\right)\right)\\ & \;=\left(\Delta_{11}^{1},\Delta_{11}^{2}\right){\left(\cos\left(\alpha_{1}+\alpha_{2}\right)\cos\frac{\theta_{1}}{2}\cos\frac{\theta_{2}}{2}+\sin\left(\beta_{1}+\beta_{2}\right)\sin\frac{\theta_{1}}{2}\sin\frac{\theta_{2}}{2}\right)}^{2}\\ \; & \;+\left(\Delta_{12}^{1},\Delta_{12}^{2}\right){\left(\cos\left(\alpha_{1}-\beta_{2}\right)\cos\frac{\theta_{1}}{2}\sin\frac{\theta_{2}}{2}+\sin\left(\alpha_{2}-\beta_{1}\right)\sin\frac{\theta_{1}}{2}\cos\frac{\theta_{2}}{2}\right)}^{2}\\ \; & \;+\left(\Delta_{21}^{1},\Delta_{21}^{2}\right){\left(\sin\left(\alpha_{1}-\beta_{2}\right)\cos\frac{\theta_{1}}{2}\sin\frac{\theta_{2}}{2}+\cos\left(\alpha_{2}-\beta_{1}\right)\sin\frac{\theta_{1}}{2}\cos\frac{\theta_{2}}{2}\right)}^{2}\\ \; & \;+\left(\Delta_{22}^{1},\Delta_{22}^{2}\right){\left(\sin\left(\alpha_{1}+\alpha_{2}\right)\cos\frac{\theta_{1}}{2}\cos\frac{\theta_{2}}{2}-\cos\left(\beta_{1}+\beta_{2}\right)\sin\frac{\theta_{1}}{2}\sin\frac{\theta_{2}}{2}\right)}^{2}. \end{array}$$

**Figure 3:**
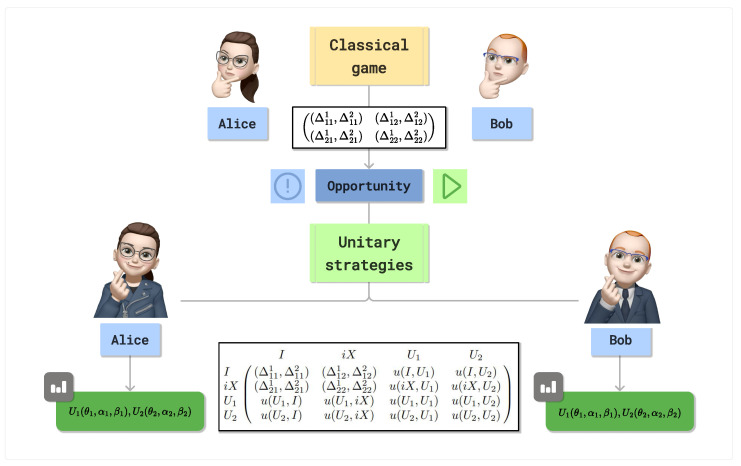
Extending classical game via EWL scheme into four-strategy quantum extension.

A classical 2×2 game is a strategic interaction between two players, each of whom selects one of two available strategies. The outcomes of these interactions are represented in a bimatrix, where each cell denotes the corresponding payoffs to both players based on their chosen strategies. Figure 3 presents a conceptual diagram illustrating how a classical game can be extended by enabling players to adopt a broader range of strategies, specifically, two unitary strategies.

Although players use all strategies in a classical manner, the quantum extensions enrich the strategic space. Even without recognizing their quantum origin, players can leverage these new options to form strategies that outperform those in the original game. It is important to emphasize that it can lead to NE that are closer to Pareto-optimal outcomes than those achievable in the purely classical setting.

In this study, we intend to utilize significant elements of John von Neumann’s utility theory. This theoretical framework offers a mechanism for classifying games. The payoff functions of all games within a class determine the same preference relations of the players. Hence, for any opponent’s strategy, the player’s optimal response remains consistent across all games within the class, making the games equivalent with respect to NE.

**Definition** **5** ([30])**.** *Let u:X→R be a function. A function v:X→R is a positive affine transformation of u if there exists a positive real number λ>0 and a real number μ such that*

(8) $$v\left(x\right)=\lambda u\left(x\right)+\mu ,\;\forall_{x\in X}.$$

A special case of von Neumann’s linear utility function theorem is

**Theorem** **1.** *If $u_{i}$ is a payoff function representing player i-th preference relation, then any positive affine transformation of $u_{i}$ is a payoff function representing the same preference relation.*

Let us consider a general PD game given by (2). Let us define a positive affine transformation of the form

(9) $$f\left(x\right)=\frac{1}{T-S}\left(x-S\right).$$

This transformation permits the payoffs of the general form of PD (2) to be reduced to two parameters, *r* and *p*, with values in the interval [0,1]:

(10) $$\begin{array}{cc} f(S) & =\frac{1}{T-S}\left(S-S\right)=0, \end{array}$$

(11) $$\begin{array}{cc} f(T) & =\frac{1}{T-S}\left(T-S\right)=1, \end{array}$$

(12) $$\begin{array}{cc} f(R) & =\frac{1}{T-S}\left(R-S\right)=r, \end{array}$$

(13) $$\begin{array}{cc} f(P) & =\frac{1}{T-S}\left(P-S\right)=p, \end{array}$$

and 0<p<r<1. As a result, we obtain a game

(14) $$(\begin{array}{cc} \left(f(R),f(R)\right) & \left(f(S),f(T)\right)\\ \left(f(T),f(S)\right) & \left(f(P),f(P)\right) \end{array}),$$

which is equivalent to game (2) with respect to preference relations. In other words, they represent the same problem from a game theory point of view. Taking into account (10)–(13) and (14) one can therefore consider a general PD game as

(15) $$\Gamma =\left(\begin{array}{cc} \left(r,r\right) & \left(0,1\right)\\ \left(1,0\right) & \left(p,p\right) \end{array}\right),\;0<p<r<1\;\mathrm{and}\;r>\frac{1}{2}.$$

**Example** **1.** *A commonly used bimatrix of the PD*

(16) $$\left(\begin{array}{cc} \left(3,3\right) & \left(0,5\right)\\ \left(5,0\right) & \left(1,1\right) \end{array}\right)$$

*is equivalent to game (15), where r=3/5 and p=1/5.*

In the remainder of this paper, we investigate NE by proving theorems about the conditions for their existence for the general form of the PD given in (15). However, for purposes of clarity, selected examples of equilibria will be presented in the context of its common form (16).

The application of a positive affine transformation in the classical game does not also affect the EWL quantization of the game.

**Proposition** **1.** *The payoffs’ preference relations of the EWL scheme are invariant with respect to a positive affine transformation of payoffs in the classical game.*

**Proof.**  Let us consider a positive affine transformation y=λx+μ and a pair of bimatrix games of the form

(17) $$\Theta_{1}=\left(\begin{array}{cc} \Delta_{11} & \Delta_{12}\\ \Delta_{21} & \Delta_{22} \end{array}\right),\;\Theta_{2}=\left(\begin{array}{cc} \lambda \Delta_{11}+\mu & \lambda \Delta_{12}+\mu \\ \lambda \Delta_{21}+\mu & \lambda \Delta_{22}+\mu \end{array}\right).$$

Let $\left(U_{1},U_{2}\right)$ be a strategy profile that is more preferred by player *i* than a profile $\left(U_{1}^{\prime },U_{2}^{\prime }\right)$. Both strategy profiles determine some probability distributions $\left(p_{kl}\right)$ and $\left(p_{kl}^{\prime }\right)$ defined by the payoff function in the EWL scheme (7) for $\Theta_{1}$, i.e., over $\left\{\Delta_{kl},k,l=1,2\right\}$, and

(18) $$\sum_{k,l=1,2}p_{kl}\Delta_{kl}^{i}\geq \sum_{k,l=1,2}p_{kl}^{\prime }\Delta_{kl}^{i}.$$

On the other hand, in the EWL scheme for $\Theta_{2}$

(19) $$\begin{array}{cc} \; & \sum_{k,l=1,2}p_{kl}\left(\lambda \Delta_{kl}^{i}+\mu \right)-\sum_{k,l=1,2}p_{kl}^{\prime }\left(\lambda \Delta_{kl}^{i}+\mu \right)\\ \; & \;=\sum_{k,l=1,2}p_{kl}\lambda \Delta_{kl}^{i}+\sum_{k,l=1,2}p_{kl}\mu -\sum_{k,l=1,2}p_{kl}^{\prime }\lambda \Delta_{kl}^{i}-\sum_{k,l=1,2}p_{kl}^{\prime }\mu \\ & \;=\lambda \left(\sum_{k,l=1,2}p_{kl}\Delta_{kl}^{i}-\sum_{k,l=1,2}p_{kl}^{\prime }\Delta_{kl}^{i}\right)\geq 0. \end{array}$$

□

Therefore, the strategy profile $\left(U_{1},U_{2}\right)$ is more preferred than a profile $\left(U_{1}^{\prime },U_{2}^{\prime }\right)$ by player *i* also in the EWL scheme of the game $\Theta_{2}$. As a result of this property, any NE found for a particular EWL quantization of game (15) will likewise serve as an equilibrium for the EWL quantization of the corresponding game (2).

## 3. Permissible Four-Strategy Quantum Extensions

The study [28] examined EWL quantizations of a 2×2 classical game by transforming it into 4×4 games, incorporating two additional unitary strategies, $U_{1}$ and $U_{2}$, alongside the classical strategies *I* and iX. It was demonstrated that there are only five classes of such quantizations that satisfy the invariance condition with respect to isomorphisms of the classical game. Such quantizations are referred to as *extensions* of the classical game. Each of the classical game extension classes below corresponds to the specific parameters $\theta_{i},\alpha_{i},\beta_{i}$, i∈{1,2} of the unitary operators $U_{1}=U_{1}\left(\theta_{1},\alpha_{1},\beta_{1}\right)$, $U_{2}=U_{2}\left(\theta_{2},\alpha_{2},\beta_{2}\right)$ of the extension. Since the focus of this paper is on NE, in the following, we will only give selected strategy parameters, e.g., those on which the payoffs of a quantum game depend. Further details regarding the remaining parameters of the strategy can be found in Table 1 of the article [28].

As demonstrated in the aforementioned paper, all four-strategy quantum extensions of the classical game defined by (15) can be expressed by the Γ matrix itself and three derivative matrices:

(20) $$\Gamma_{1}=\left(\begin{array}{cc} \left(1,0\right) & \left(p,p\right)\\ \left(r,r\right) & \left(0,1\right) \end{array}\right),\;\Gamma_{2}=\left(\begin{array}{cc} \left(0,1\right) & \left(r,r\right)\\ \left(p,p\right) & \left(1,0\right) \end{array}\right),\;\Gamma_{3}=\left(\begin{array}{cc} \left(p,p\right) & \left(1,0\right)\\ \left(0,1\right) & \left(r,r\right) \end{array}\right),$$

derived from (15), by swapping rows, columns, or both.

The first extension class *A* is defined by matrices

(21) $$A_{1}=\left(\begin{array}{cc} \Gamma & a_{1}\Gamma +a_{1}^{\prime }\Gamma_{3}\\ a_{1}\Gamma +a_{1}^{\prime }\Gamma_{3} & b_{1}\Gamma +b_{1}^{\prime }\Gamma_{3} \end{array}\right),\;A_{2}=\left(\begin{array}{cc} \Gamma & a_{2}\Gamma_{2}+a_{2}^{\prime }\Gamma_{1}\\ a_{2}\Gamma_{1}+a_{2}^{\prime }\Gamma_{2} & b_{2}\Gamma_{3}+b_{2}^{\prime }\Gamma \end{array}\right),$$

where $a_{i}=\cos^{2}\alpha_{i}$, $a_{i}^{\prime }=1-a_{i}=\sin^{2}\alpha_{i}$ and $b_{i}=\cos^{2}2\alpha_{i}$, $b_{i}^{\prime }=1-b_{i}=\sin^{2}2\alpha_{i}$. Other parameters of quantum strategies are defined in [28], in particular $\theta_{1}=0$ and $\theta_{2}=\pi$ for $A_{1}$ and vice versa for $A_{2}$. The second class of extensions *B*, where $\theta_{1}=\theta_{2}=\frac{\pi }{2}$, is characterized by the matrix

(22) $$B=(\begin{array}{cc} \Gamma & \frac{\Gamma +\Gamma_{1}+\Gamma_{2}+\Gamma_{3}}{4}\\ \frac{\Gamma +\Gamma_{1}+\Gamma_{2}+\Gamma_{3}}{4} & \frac{\Gamma +\Gamma_{1}+\Gamma_{2}+\Gamma_{3}}{4} \end{array}).$$

Extension of the class *C* is given by the formula

(23) $$C=(\begin{array}{cc} \Gamma & t\frac{\Gamma +\Gamma_{3}}{2}+t^{\prime }\frac{\Gamma_{1}+\Gamma_{2}}{2}\\ t\frac{\Gamma +\Gamma_{3}}{2}+t^{\prime }\frac{\Gamma_{1}+\Gamma_{2}}{2} & t^{\prime 2}\Gamma +tt^{\prime }\left(\Gamma_{1}+\Gamma_{2}\right)+t^{2}\Gamma_{3} \end{array}),$$

where $t=\cos^{2}\frac{\theta_{1}}{2}$, $t^{\prime }=1-t=\sin^{2}\frac{\theta_{1}}{2}$. For class *C*, as well as for classes *D* and *E*, $\theta_{2}=\pi -\theta_{1}$. The class *D* can be determined by the following matrices:

(24) $$\begin{array}{cc} \; & D_{1}=\left(\begin{array}{cc} \Gamma & t\Gamma +t^{\prime }\Gamma_{2}\\ t\Gamma +t^{\prime }\Gamma_{1} & t^{2}\Gamma +tt^{\prime }\left(\Gamma_{1}+\Gamma_{2}\right)+t^{\prime 2}\Gamma_{3} \end{array}\right),\\ \; & D_{2}=\left(\begin{array}{cc} \Gamma & t\Gamma_{3}+t^{\prime }\Gamma_{1}\\ t\Gamma_{3}+t^{\prime }\Gamma_{2} & t^{2}\Gamma +tt^{\prime }\left(\Gamma_{1}+\Gamma_{2}\right)+t^{\prime 2}\Gamma_{3} \end{array}\right). \end{array}$$

The last class *E* is determined by the matrices

(25) $$\begin{array}{cc} \; & E_{1}=\left(\begin{array}{cc} \Gamma & t\Gamma +t^{\prime }\Gamma_{1}\\ t\Gamma +t^{\prime }\Gamma_{2} & t^{2}\Gamma +tt^{\prime }\left(\Gamma_{1}+\Gamma_{2}\right)+t^{\prime 2}\Gamma_{3} \end{array}\right),\\ \; & E_{2}=\left(\begin{array}{cc} \Gamma & t\Gamma_{3}+t^{\prime }\Gamma_{2}\\ t\Gamma_{3}+t^{\prime }\Gamma_{1} & t^{2}\Gamma +tt^{\prime }\left(\Gamma_{1}+\Gamma_{2}\right)+t^{\prime 2}\Gamma_{3} \end{array}\right). \end{array}$$

The analysis of NE will be simplified by the symmetry of the extension matrix. Consequently, we will prove the following theorem.

**Proposition** **2.** *If a two-player game *Γ* is symmetric, then its quantum EWL extension is also a symmetric game.*

**Proof.**  First note that

(26) $$|\langle \psi_{kl}|U_{2}⊗U_{1}|\psi_{11}{\rangle |}^{2}=|\langle \psi_{lk}|U_{1}⊗U_{2}|\psi_{11}{\rangle |}^{2}$$

in Formula (6) for each pair $\left(k,l\right)\in {\left\{1,2\right\}}^{2}$. Moreover, if a bimatrix game Γ is symmetric then $\Delta_{ij}^{2}=\Delta_{ji}^{1}$. Then, it follows that

(27) $$\begin{array}{cc} u_{2}\left(U_{2},U_{1}\right) & =\sum_{k,l\in \{1,2\}}\Delta_{kl}^{2}|\langle \psi_{kl}|U_{2}⊗U_{1}|\psi_{11}{\rangle |}^{2}\\ & =\sum_{k,l\in \{1,2\}}\Delta_{lk}^{1}|\langle \psi_{lk}|U_{1}⊗U_{2}|\psi_{11}{\rangle |}^{2}=u_{1}\left(U_{1},U_{2}\right). \end{array}$$

□

Based on Proposition 2, the following conclusion can be drawn:

**Corollary** **1.** *If a two-player game *Γ* is symmetric, then all extensions $A_{1},\ldots ,E_{2}$ are also symmetric games.*

**Example** **2.** *As an example, let us examine the symmetries of the *Γ* game (15):*

(28) $$\Gamma =\left(\begin{array}{cc} \left(r,r\right) & \left(0,1\right)\\ \left(1,0\right) & \left(p,p\right) \end{array}\right)=\left(\Gamma^{1},\Gamma^{2}\right).$$

*It is symmetric, as the players’ payoffs submatrices $\Gamma^{i}$ obey the relation*

(29) $$\Gamma^{2}=\left(\begin{array}{cc} r & 1\\ 0 & p \end{array}\right)={\left(\Gamma^{1}\right)}^{T}.$$

*In addition, definition (20) allows us to infer that*

(30) $$\Gamma_{1}^{2}=\left(\begin{array}{cc} 0 & p\\ r & 1 \end{array}\right)={\left(\Gamma_{2}^{1}\right)}^{T},\;\Gamma_{2}^{1}=\left(\begin{array}{cc} 0 & r\\ p & 1 \end{array}\right)={\left(\Gamma_{1}^{2}\right)}^{T}\;and\;\Gamma_{3}^{2}=\left(\begin{array}{cc} p & 0\\ 1 & r \end{array}\right)$$

*The symmetry of the extension matrices (21) and (22)–(25) can be attributed to the relationships given in (29) and (30). To illustrate, consider the extension $A_{2}$:*

(31) $$\begin{array}{cc} {\left(A_{2}^{1}\right)}^{T} & ={\left(\begin{array}{cc} \Gamma^{1} & a_{2}\Gamma_{2}^{1}+a_{2}^{\prime }\Gamma_{1}^{1}\\ a_{2}\Gamma_{1}^{1}+a_{2}^{\prime }\Gamma_{2}^{1} & b_{2}\Gamma_{3}^{1}+b_{2}^{\prime }\Gamma^{1} \end{array}\right)}^{T}\\ & =(\begin{array}{cc} {\left(\Gamma^{1}\right)}^{T} & {\left(a_{2}\Gamma_{1}^{1}+a_{2}^{\prime }\Gamma_{2}^{1}\right)}^{T}\\ {\left(a_{2}\Gamma_{2}^{1}+a_{2}^{\prime }\Gamma_{1}^{1}\right)}^{T} & {\left(b_{2}\Gamma_{3}^{1}+b_{2}^{\prime }\Gamma^{1}\right)}^{T} \end{array})\\ & =\left(\begin{array}{cc} \Gamma^{2} & a_{2}\Gamma_{2}^{2}+a_{2}^{\prime }\Gamma_{1}^{2}\\ a_{2}\Gamma_{1}^{2}+a_{2}^{\prime }\Gamma_{2}^{2} & b_{2}\Gamma_{3}^{2}+b_{2}^{\prime }\Gamma^{2} \end{array}\right)=A_{2}^{2}. \end{array}$$

Throughout the rest of this analysis, to simplify the equations, our attention will be centered on the extension matrices of the first player. It is understood that the matrices for the second player are simply the transposed versions of these.

## 4. Nash Equilibria of the Quantum Extensions of the Prisoner’s Dilemma

This section aims to conduct a thorough analysis of all PD extensions to identify NE in pure strategies. For each equilibrium, we will show the necessary conditions that must be met by the payoffs *r* and *p* of the general PD (15), as well as the parameters $\theta_{i}$ or $\alpha_{i}$ associated with the quantum strategies (3). For a specified extension, the parameters $\beta_{i}$ of the quantum strategy are each time determined by the parameters $\alpha_{i}$ [28].

### 4.1. Extension of the *A* Class

Let $A_{1}=\left(A_{1}^{1},{\left(A_{1}^{1}\right)}^{T}\right)$, where

(32) $$A_{1}^{1}=\left(\begin{array}{cccc} r & 0 & a_{1}r+a_{1}^{\prime }p & a_{1}^{\prime }\\ 1 & p & a_{1} & a_{1}p+a_{1}^{\prime }r\\ a_{1}r+a_{1}^{\prime }p & a_{1}^{\prime } & b_{1}r+b_{1}^{\prime }p & b_{1}^{\prime }\\ a_{1} & a_{1}p+a_{1}^{\prime }r & b_{1} & b_{1}p+b_{1}^{\prime }r \end{array}\right).$$

The parameters $a_{i},a_{i}^{\prime },b_{i},$ and $b_{i}^{\prime }$, previously defined for i=1,2, can each be represented in terms of the single parameter *a*:

(33) $$\begin{array}{cc} \; & a_{i}=\cos^{2}\left(\alpha_{i}\right)=a,\\ \; & a_{i}^{\prime }=\sin^{2}\left(\alpha_{i}\right)=1-a,\\ \; & b_{i}=\cos^{2}\left(2\alpha_{i}\right)={\left(1-2a\right)}^{2},\\ \; & b_{i}^{\prime }=\sin^{2}\left(2\alpha_{i}\right)=4a\left(1-a\right). \end{array}$$

Note that $\alpha_{i}\in \left[0,2\pi \right)$ corresponds to a∈[0,1]. This shortened notation will remain clear, assuming we keep in mind that the parameter $a=a_{i}$ is consistently present in the extension $A_{i}$. As a result, $A_{1}^{1}$ matrix takes the following form:

(34) $$\begin{array}{c} A_{1}^{1}=\left(\begin{array}{cccc} r & 0 & ar-ap+p & 1-a\\ 1 & p & a & ap-ar+r\\ ar-ap+p & 1-a & r-4(a-1)a(p-r) & -4(a-1)a\\ a & ap-ar+r & {\left(1-2a\right)}^{2} & {\left(1-2a\right)}^{2}p-4\left(a-1\right)ar \end{array}\right). \end{array}$$

Propositions 3–9 demonstrate the existence of potential Nash equilibria (NE) for sequential profile strategies of extension $A_{1}$ and specify the conditions required for their presence. Proofs of all these propositions can be found in the appendices.

**Proposition** **3.** *Neither (1,j) nor (i,1), i,j=1,…,4 are Nash equilibria.*

**Proposition** **4.** *The strategy profile (2,2) is a Nash equilibrium for 0<p<r,$\frac{1}{2}<r<1$, provided a=1.*

**Proposition** **5.** *The strategy profiles (2,3) and (3,2) represent Nash equilibria if any one of the following four conditions is met:*

(35) $$0<p\leq \frac{1}{6}\wedge \frac{1}{2}<r\leq 1-3p\wedge \frac{1}{4}\leq a\leq \frac{r-1}{r-1-p}$$

*or*

(36) $$0<p\leq \frac{1}{6}\wedge 1-3p<r<1-p\wedge \frac{p}{1+p-r}\leq a\leq \frac{r-1}{r-1-p}$$

*or*

(37) $$0<p\leq \frac{1}{6}\wedge r=1-p\wedge a=\frac{r-1}{r-1-p}$$

*or*

(38) $$\frac{1}{6}<p<\frac{1}{2}\wedge \frac{1}{2}<r\leq 1-p\wedge \frac{p}{1+p-r}\leq a\leq \frac{r-1}{r-1-p}.$$

*Note that if r=1−p in Equation (38), then $a=\frac{r-1}{r-1-p}$.*

**Proposition** **6.** *The strategy profiles (2,4) and (4,2) are Nash equilibria given that*

(39) $$\begin{array}{c} \left(\frac{1}{2}<r<\frac{3-p}{3}\wedge a=1\right)\vee \left(r=\frac{3-p}{3}\wedge a\in \left\{\frac{1}{4},1\right\}\right)\\ \vee \left(\frac{3-p}{3}<r<1\wedge a\in \left[\frac{1-r}{1+p-r},1\right]\right). \end{array}$$

**Proposition** **7.** *The strategy profile (3,3) represents a Nash equilibrium provided that*

(40) $$\begin{array}{c} 0<p<\frac{1}{6}\wedge \left(\left(r=1-3p\wedge a=\frac{1}{4}\right)\vee \left(1-3p<r<1\wedge \frac{1}{2}-\frac{1}{2}\sqrt{\frac{p}{1+p-r}}\leq a\leq \frac{1}{4}\right)\right) \end{array}$$

*or*

(41) $$\frac{1}{6}\leq p\leq \frac{1}{2}\wedge \frac{1}{2}<r<1\wedge \frac{1}{2}-\frac{1}{2}\sqrt{\frac{p}{1+p-r}}\leq a\leq \frac{1}{4}$$

*or*

(42) $$\frac{1}{2}<p<r\wedge p<r<1\wedge \frac{1}{2}-\frac{1}{2}\sqrt{\frac{p}{1+p-r}}\leq a\leq \frac{1}{4}.$$

**Proposition** **8.** *The strategy profiles (3,4) and (4,3) are Nash equilibria if $a=\frac{1}{4}$, $\frac{1}{2}<r\leq 1-3p$, and $0<p<\frac{1}{6}$.*

**Proposition** **9.** *The strategy profile (4,4) is a Nash equilibrium under the condition that at least one of the following criteria is met:*

(43) $$\frac{1}{2}<r\leq \frac{3}{4}\wedge 0<p<r\wedge \frac{1}{2}+\frac{1}{2}\sqrt{\frac{1-r}{p-r+1}}\leq a\leq 1$$

*or*

(44) $$\frac{3}{4}<r<1\wedge 0<p<3-3r\wedge \frac{1}{2}+\frac{1}{2}\sqrt{\frac{1-r}{p-r+1}}\leq a\leq 1$$

*or*

(45) $$\frac{3}{4}<r<1\wedge p=3-3r\wedge \left(a=\frac{1}{4}\vee \frac{1}{2}+\frac{1}{2}\sqrt{\frac{1-r}{p-r+1}}\leq a\leq 1\right)$$

*or*

(46) $$\begin{array}{c} \frac{3}{4}<r<1\wedge 3-3r<p<r\\ \wedge \left(\frac{1}{4}\leq a\leq \frac{1}{2}-\frac{1}{2}\sqrt{\frac{1-r}{p-r+1}}\vee \frac{1}{2}+\frac{1}{2}\sqrt{\frac{1-r}{p-r+1}}\leq a\leq 1\right). \end{array}$$

Observe that the matrix $A_{2}$ is derived from the matrix $A_{1}$ by swapping the third and fourth rows and columns. Consequently, an analogous set of Propositions 3–9, describing NE, can be demonstrated for the extensions of $A_{2}$. Table 1 presents a compilation of all strategy profiles in the extensions of $A_{1}$ and $A_{2}$ where NE can exist, along with the conditions for the payoffs *p* and *r*, and the parameter *a*. In the subsequent example, we present the NE of the $A_{1}$ extension of the PD in its standard form (16).

**Example** **3.** *Consider the PD given by Equation (16). Below is the resulting matrix for the $A_{1}$ class extension.*

(47) $$\begin{array}{c} A_{1}=\left(\begin{array}{cccc} \left(3,3\right) & \left(0,5\right) & \left(2a+1,2a+1\right) & \left(5-5a,5a\right)\\ \left(5,0\right) & \left(1,1\right) & \left(5a,5-5a\right) & \left(3-2a,3-2a\right)\\ \left(2a+1,2a+1\right) & \left(5-5a,5a\right) & \left(8(a-1)a+3,8(a-1)a+3\right) & \left(-20\left(a-1\right)a,5{\left(1-2a\right)}^{2}\right)\\ \left(5a,5-5a\right) & \left(3-2a,3-2a\right) & \left(5{\left(1-2a\right)}^{2},-20\left(a-1\right)a\right) & \left(1-8(a-1)a,1-8(a-1)a\right) \end{array}\right). \end{array}$$

*It should be observed that for strategy profiles (3,4) and (4,3), the requisite condition $0<p<\frac{1}{6}$ from Proposition 8, which is essential for the existence of an NE, is not met because $p=\frac{1}{5}$. Table 2 illustrates seven strategy profiles for which NE are feasible, along with the corresponding values of the parameter a that result in maximum equal payoffs for the players.*

**Figure 4:**
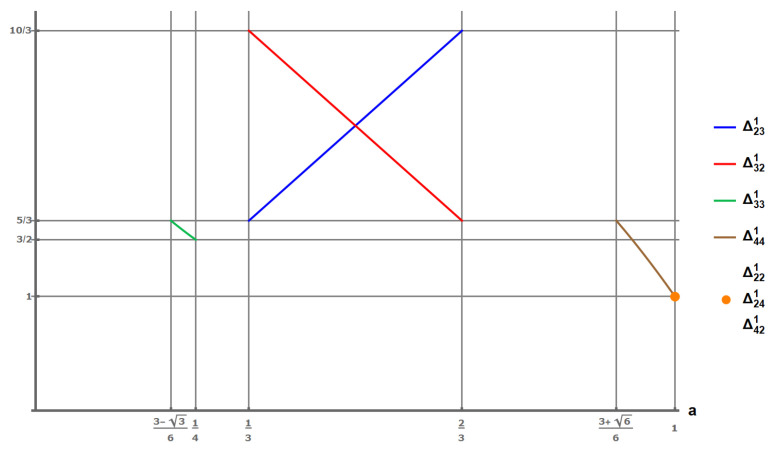
The dependence of NE first-player payoffs on the value of the parameter a (in the permissible range) for different strategy profiles of the exemplary PD (16) in the extension $A_{1}$ given by matrix (47). Payoffs $\Delta_{22}^{1}=\Delta_{24}^{1}=\Delta_{42}^{1}=1$, which correspond to NE for a=1 are identical and depicted by a single dot.

*Figure 4 shows the first player’s payoffs for all NE (not necessarily with equal payoffs) of the $A_{1}$ extension of the PD (16) as a function of the parameter a. The maximum total payoff of players is equal to 5, i.e., T+S, when $\frac{1}{3}<a<\frac{2}{3}$. The maximum equal payoff is $\frac{5}{2}$, achievable at $a=\frac{1}{2}$.*

**Figure 5:**
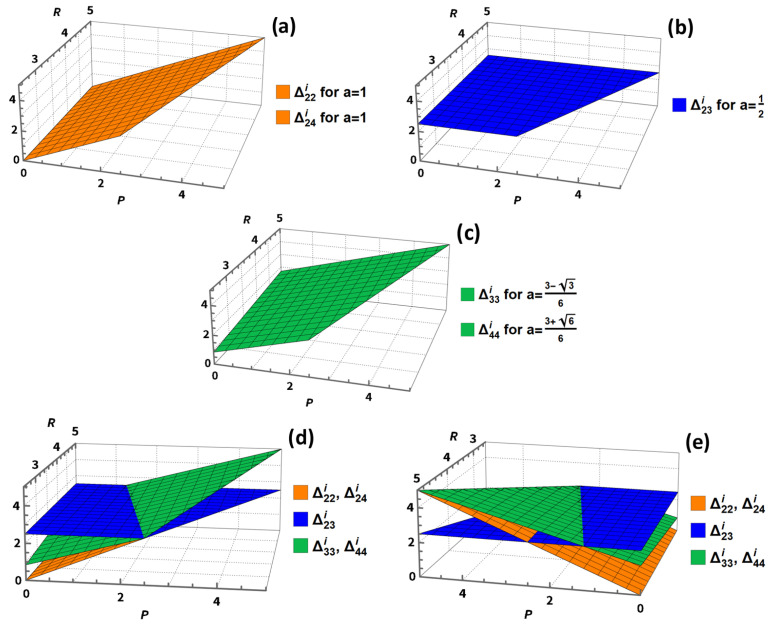
Dependence of the payoffs of the $A_{1}$ extension of the PD (2) on the payoffs *P* and *R* for S=0 and T=5 and the value of *a* corresponding to the maximum and equal NE according to Table 2. For a better comparison, figures (**d**,**e**) show the relationships shown in (**a**–**c**) from two different points of view. In all presented cases the payoffs $\Delta_{jk}^{i}$ are the same for both players i∈{1,2}.

Figure 5 shows the payoffs $\Delta_{jk}^{i}$ for profiles j≤k of NE in the $A_{1}$ extension of the PD (2) as a function of the payoffs *P* and *R* for S=0 and T=5 and the value of *a* corresponding to the maximum and equal NE according to Table 2.

**Figure 6:**
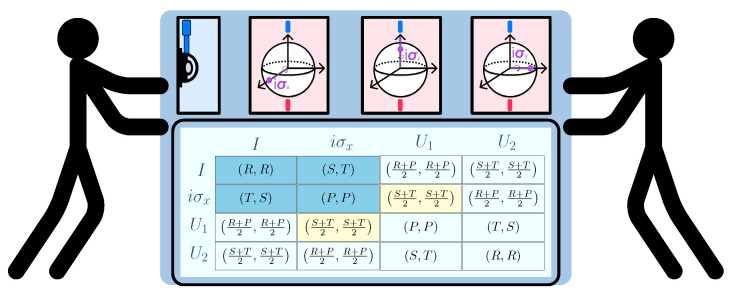
Class $A_{1}$ quantum extension as detailed in Table 1 has Nash equilibria at (2,3) and (3,2) (highlighted in yellow) that are more aligned with Pareto optimality compared to the classical PD (dark blue). In this quantum setting, players can choose one ‘Cooperate’ strategy, denoted *I*, alongside three ‘Defect’ strategies, represented by the Pauli matrix $i\sigma_{x}$, as well as the following linear combinations, namely $U_{1}=\frac{I+i\sigma_{z}}{\sqrt{2}}$ and $U_{2}=\frac{i\sigma_{x}+i\sigma_{y}}{\sqrt{2}}$.

Figure 6 demonstrates the solution to the Nash equilibrium Pareto optimality issue for the PD within the $A_{1}$ class extension. In accordance with Table 1, the strategy profiles (2,3) and (3,2) become NE for $p\in (\frac{1}{6},\frac{1}{2})$ and $r\in (\frac{1}{2},1-p)$, which is the case for the standard PD form (16). If, in addition, a=1/2, these equilibria assume values that are both equal to and closer to Pareto-optimal solutions than *P*. It is noteworthy that within the quantum game framework, the classical ‘Cooperate’ strategy is equivalent to the identity transformation *I*, while the classical ‘Defect’ strategy corresponds to the Pauli matrix $i\sigma_{x}$. But, the quantum game introduces two additional ’Defect’ strategies, which are represented by linear combinations of the Pauli matrices, specifically $U_{1}=\frac{I+i\sigma_{z}}{\sqrt{2}}$ and $U_{2}=\frac{i\sigma_{x}+i\sigma_{y}}{\sqrt{2}}$ [28]. Participants involved in such a quantum extension of the Prisoner’s Dilemma are not required to be aware that they are engaging in a quantum variant of the game. They can attain an improved NE simply by selecting among the available strategies in a classical manner. While mutual cooperation remains the optimal strategy, the presence of three defecting strategies per player leads to a superior NE in quantum settings compared to classical scenarios. In addition, by adjusting the players’ strategies by altering the parameter *a* within the interval $\frac{1}{3}<a<\frac{2}{3}$ (see Figure 4), the payoffs for the strategy profiles (2,3) and (3,2) mimic those of a battle of the sexes game in this specified range.

### 4.2. Extension of the *B* Class

The *B* class extension of PD (15) is defined by the first player’s payoff matrix:

(48) $$B^{1}=\left(\begin{array}{cccc} r & 0 & \frac{1}{4}\left(1+r+p\right) & \frac{1}{4}\left(1+r+p\right)\\ 1 & p & \frac{1}{4}\left(1+r+p\right) & \frac{1}{4}\left(1+r+p\right)\\ \frac{1}{4}\left(1+r+p\right) & \frac{1}{4}\left(1+r+p\right) & \frac{1}{4}\left(1+r+p\right) & \frac{1}{4}\left(1+r+p\right)\\ \frac{1}{4}\left(1+r+p\right) & \frac{1}{4}\left(1+r+p\right) & \frac{1}{4}\left(1+r+p\right) & \frac{1}{4}\left(1+r+p\right) \end{array}\right),$$

where 0<p<r<1 and 2r>1.

**Proposition** **10.** *Depending on the parameters p and r, the game defined by matrix (48) exhibits the following Nash equilibria in pure strategies:*

*i*. *The strategy profiles (1,j) and (i,1), for i,j=1,…,4 are not NE for any values of p and r.*
*ii*. *The strategy profile (2,2) is an NE provided that $p\geq \frac{1+r}{3}$.*
*iii*. *The strategy profile (2,j) and (i,2) for i,j=3,4 are NE provided that $p\leq \frac{1+r}{3}$.*
*iv*. *The strategy profiles (i,j), for i,j=3,4 are NE for arbitrary values of p and r.*

The above proposition is summarised in Table 3, which shows the conditions that must be met for NE to exist in the respective pure strategy profiles. The payoff values for these equilibria are the same for both players and equal to the corresponding positions of matrix (48).

**Example** **4.** *For the standard PD payoff matrix (16), the equivalent game (15) parameters are r=3/5 and p=1/5; therefore $p<\frac{1+r}{3}$. This leads to the set of NE strategy profiles {(i,j):i≥3∨j≥3} with payoffs all equal to $2\frac{1}{4}$; see Table 4.*

### 4.3. Extension of the *C* Class

In this subsection, we examine the extension of class *C* to analyze the potential NE. The payoff matrix for the first player is

(49) $$\begin{array}{c} C^{1}=\left(\begin{array}{cccc} r & 0 & \frac{t}{2}\left(p+r\right)+\frac{1-t}{2} & \frac{1}{2}\left(1-t\right)\left(p+r\right)+\frac{t}{2}\\ 1 & p & \frac{1}{2}\left(1-t\right)\left(p+r\right)+\frac{t}{2} & \frac{t}{2}\left(p+r\right)+\frac{1-t}{2},\\ \frac{t}{2}\left(p+r\right)+\frac{1-t}{2} & \frac{1}{2}\left(1-t\right)\left(p+r\right)+\frac{t}{2} & pt^{2}+r{\left(1-t\right)}^{2}+t\left(1-t\right) & \left(1-t\right)t\left(p+r\right)+t^{2}\\ \frac{1}{2}\left(1-t\right)\left(p+r\right)+\frac{t}{2} & \frac{t}{2}\left(p+r\right)+\frac{1-t}{2} & t\left(1-t\right)\left(p+r\right)+{\left(1-t\right)}^{2} & p{\left(1-t\right)}^{2}+rt^{2}+t\left(1-t\right) \end{array}\right). \end{array}$$

In this case, the existence of NE is dependent on *p* and *r*, namely, the PD payoffs, and the EWL scheme parameter *t*, as outlined in (23). Similarly to previous sections, the NE for consecutive strategy profiles is articulated in theorems, with their proofs provided in the appendices. Neither the pair of strategies in the first row nor those in the first column of class *C* can lead to an NE. This is substantiated by the following proposition.

**Proposition** **11.** *The pair (1,j) and the pair (i,1), where i,j∈{1,2,3,4}, are not Nash equilibria.*

The subsequent propositions outline the necessary conditions for the parameters *t*, *p*, and *r* to ensure the existence of NE within the remaining diagonal strategy profiles.

**Proposition** **12.** *The strategy profile (2,2) is a Nash equilibrium when either the inequality $\frac{r-p}{p+r-1}\leq t\leq \frac{2p-1}{p+r-1}$ with $p>\frac{1}{2}$ is satisfied, or in the case where $t=\frac{1}{2}$ and $p=\frac{1+r}{3}$.*

**Proposition** **13.** *The strategy profile (3,3) is a Nash equilibrium provided that $t=\frac{1}{2}$.*

**Proposition** **14.** *The strategy profile (4,4) is a Nash equilibrium provided that $t=\frac{1}{2}$.*

To demonstrate under which conditions the remaining off-diagonal strategy profiles are NE of the *C* class game, the following proposition can be proven.

**Proposition** **15.** *Let 0<p<1−r. Strategy profiles (3,2) and (2,3) are Nash equilibria if $t\geq \frac{1}{2}$. Moreover, (3,2) and (2,3) are Nash equilibria if $t=\frac{1}{2}$ and $1-r<p<\frac{1+r}{3}$.*

**Proposition** **16.** *Let 0<p<1−r. Pairs of strategies (4,2) and (2,4) are Nash equilibria if $t\leq \frac{1}{2}$. In particular, (4,2) and (2,4) are Nash equilibria if $t=\frac{1}{2}$ and $1-r<p<\frac{1+r}{3}$.*

**Proposition** **17.** *Strategy profiles (3,4) and (4,3) are Nash equilibria provided that $t=\frac{1}{2}$.*

The existence of NE in the *C* class extension can be summarized in the following Table 5, where particular cells refer to the corresponding strategy profiles of the *C* class.

**Example** **5.** *The extension of the C class in the context of the PD for commonly encountered payoffs (16) takes the following form:*

(50) $$\begin{array}{cc} & C=(\begin{array}{cccc} \left(3,3\right) & \left(0,5\right) & \left(\frac{5-t}{2},\frac{5-t}{2}\right) & \left(\frac{4+t}{2},\frac{4+t}{2}\right)\\ \left(5,0\right) & \left(1,1\right) & \left(\frac{4+t}{2},\frac{4+t}{2}\right) & \left(\frac{5-t}{2},\frac{5-t}{2}\right)\\ \left(\frac{5-t}{2},\frac{5-t}{2}\right) & \left(\frac{4+t}{2},\frac{4+t}{2}\right) & \left(3-t-t^{2},3-t-t^{2}\right) & \left(t\left(t+4\right),5-6t+t^{2}\right)\\ \left(\frac{4+t}{2},\frac{4+t}{2}\right) & \left(\frac{5-t}{2},\frac{5-t}{2}\right) & \left(5-6t+t^{2},t\left(t+4\right)\right) & \left(-t^{2}+3t+1,-t^{2}+3t+1\right) \end{array}) \end{array}$$

*In this version of PD, it is important to observe that the strategy profile (2,2) does not fulfill the requirement $p>\frac{1}{2}$ because here $p=\frac{1}{5}$. Consequently, (2,2) does not represent an NE. Therefore, there are eight pure NE denoted graphically in Table 6. The maximum payoff for both players is equal to $2\frac{1}{2}$ and is achieved for two pairs of strategies (2,3) and (3,2). It is noteworthy that the highest payoff for these two strategic pairs is achieved at the upper threshold of t, which in this instance is 1 (refer to Table 5).*

*Proposition 12 demonstrates that for the strategy profile (2,2) to be an NE, it is adequate to assume r=4/5 and p=3/5. This pair of payoff values meets the criterion p=(r+1)/3 and constitutes an NE when t=1/2. For t=1/2, the extension of the C class defined by Equation (49) is equivalent to the B class extension [28] as expressed in Equation (48).*

### 4.4. Extension of the *D* Class

Let $D_{1}=\left(D_{1}^{1},{\left(D_{1}^{1}\right)}^{T}\right)$. The payoff matrix for the first player is

(51) $$\begin{array}{c} D_{1}^{1}=(\begin{array}{cccc} r & 0 & rt & r-rt\\ 1 & p & (1-p)t+p & (p-1)t+1\\ (r-1)t+1 & p-pt & \left(r-1\right)t^{2}+t+p{\left(1-t\right)}^{2} & \left(p+r\right)\left(t-t^{2}\right)+{\left(1-t\right)}^{2}\\ (1-r)t+r & pt & \left(1-p-r\right)t^{2}+\left(p+r\right)t & \left(p-1\right)t^{2}+t+r{\left(1-t\right)}^{2} \end{array}). \end{array}$$

**Proposition** **18.** *Consider t∈(0,1). The strategy profile (2,2) represents the sole Nash equilibrium in the $D_{1}$ extension, irrespective of values for p and r.*

**Example** **6.** *Consider the standard version of the Prisoner’s Dilemma as represented in (16). The corresponding $D_{1}$ extension is given by*

(52) $$\begin{array}{c} D_{1}=\left(\begin{array}{cccc} \left(3,3\right) & \left(0,5\right) & \left(3t,5-2t\right) & \left(3-3t,2t+3\right)\\ \left(5,0\right) & \left(1,1\right) & \left(4t+1,1-t\right) & \left(5-4t,t\right)\\ \left(5-2t,3t\right) & \left(1-t,4t+1\right) & \left(1-t^{2}+3t,1-t^{2}+3t\right) & \left(t^{2}-6t+5,t^{2}+4t\right)\\ \left(2t+3,3-3t\right) & \left(t,5-4t\right) & \left(t^{2}+4t,t^{2}-6t+5\right) & \left(3-t^{2}-t,3-t^{2}-t\right) \end{array}\right) \end{array}$$

*which maintains a single pure NE at the strategy profile (2,2), identical to the classic PD.*

Conversely, if you examine the extension of $D_{2}=\left(D_{2}^{1},{\left(D_{2}^{1}\right)}^{T}\right)$, where

(53) $$\begin{array}{c} D_{2}^{1}=\left(\begin{array}{cccc} r & 0 & (p-1)t+1 & (1-p)t+p\\ 1 & p & r-rt & rt\\ pt & (1-r)t+r & \left(r-1\right)t^{2}+t+p{\left(1-t\right)}^{2} & \left(p+r\right)\left(t-t^{2}\right)+{\left(1-t\right)}^{2}\\ p-pt & (r-1)t+1 & \left(1-p-r\right)t^{2}+\left(p+r\right)t & \left(p-1\right)t^{2}+t+r{\left(1-t\right)}^{2} \end{array}\right). \end{array}$$

**Proposition** **19.** *The game $D_{2}$ does not have Nash equilibria in pure strategies.*

**Example** **7.** *Thus, the $D_{2}$ extension of the standard Prisoner’s Dilemma (16)*

(54) $$\begin{array}{c} D_{2}=\left(\begin{array}{cccc} \left(3,3\right) & \left(0,5\right) & \left(5-4t,t\right) & \left(1+4t,1-t\right)\\ \left(5,0\right) & \left(1,1\right) & \left(3-3t,3+2t\right) & \left(3t,5-2t\right)\\ \left(t,5-4t\right) & \left(3+2t,3-3t\right) & \left(1-t^{2}+3t,1-t^{2}+3t\right) & \left(t^{2}-6t+5,t^{2}+4t\right)\\ \left(1-t,1+4t\right) & \left(5-2t,3t\right) & \left(t^{2}+4t,t^{2}-6t+5\right) & \left(3-t^{2}-t,3-t^{2}-t\right) \end{array}\right), \end{array}$$

*has no NE in pure strategies for any values of t.*

### 4.5. Extension of the *E* Class

The first player’s payoff matrix for $E_{1}=\left(E_{1}^{1},{\left(E_{1}^{1}\right)}^{T}\right)$ is

(55) $$\begin{array}{c} E_{1}^{1}=\left(\begin{array}{cccc} r & 0 & (r-1)t+1 & p-pt\\ 1 & p & (1-r)t+r & pt\\ rt & r-rt & \left(r-1\right)t^{2}+t+p{\left(1-t\right)}^{2} & \left(p+r\right)\left(t-t^{2}\right)+{\left(1-t\right)}^{2}\\ (1-p)t+p & (p-1)t+1 & \left(1-p-r\right)t^{2}+\left(p+r\right)t & \left(p-1\right)t^{2}+t+r{\left(1-t\right)}^{2} \end{array}\right). \end{array}$$

**Proposition** **20.** *Let $\frac{1}{2}\leq t<1$. Then a strategy profile (4,4) is a sole Nash equilibrium of $E_{1}$ game for all p, r.*

**Example** **8.** *Consider the PD (16). Then*

(56) $$\begin{array}{c} E_{1}=\left(\begin{array}{cccc} \left(3,3\right) & \left(0,5\right) & \left(5-2t,3t\right) & \left(1-t,4t+1\right)\\ \left(5,0\right) & \left(1,1\right) & \left(2t+3,3-3t\right) & \left(t,5-4t\right)\\ \left(3t,5-2t\right) & \left(3-3t,2t+3\right) & \left(1-t^{2}+3t,1-t^{2}+3t\right) & \left(t^{2}-6t+5,t^{2}+4t\right)\\ \left(4t+1,1-t\right) & \left(5-4t,t\right) & \left(t^{2}+4t,t^{2}-6t+5\right) & \left(3-t^{2}-t,3-t^{2}-t\right) \end{array}\right). \end{array}$$

*There exists a unique pure NE at the strategy profile (4,4). The highest possible payoff for each player occurs when t=1/2, yielding an equivalent payoff of $2\frac{1}{4}$ for both participants.*

Let $E_{2}=\left(E_{2}^{1},{\left(E_{2}^{1}\right)}^{T}\right)$, where

(57) $$\begin{array}{c} E_{2}^{1}=\left(\begin{array}{cccc} r & 0 & pt & (1-r)t+r\\ 1 & p & p-pt & (r-1)t+1\\ (p-1)t+1 & (1-p)t+p & \left(r-1\right)t^{2}+t+p{\left(1-t\right)}^{2} & \left(p+r\right)\left(t-t^{2}\right)+{\left(1-t\right)}^{2}\\ r-rt & rt & \left(1-p-r\right)t^{2}+\left(p+r\right)t & \left(p-1\right)t^{2}+t+r{\left(1-t\right)}^{2} \end{array}\right). \end{array}$$

**Proposition** **21.** *Let $0<t\leq \frac{1}{2}$. Then a strategy profile (3,3) is a sole Nash equilibrium of game $E_{2}$.*

A summary of all strategy profiles in the extensions of *D* and *E* for which NE are possible, along with the requirements for payoffs *p* and *r*, and the parameter *t*, is shown in Table 7.

**Example** **9.** *Consider the PD (16). Then*

(58) $$\begin{array}{c} E_{2}=\left(\begin{array}{cccc} \left(3,3\right) & \left(0,5\right) & \left(t,5-4t\right) & \left(2t+3,3-3t\right)\\ \left(5,0\right) & \left(1,1\right) & \left(1-t,4t+1\right) & \left(5t-2,3t\right)\\ \left(5-4t,t\right) & \left(4t+1,1-t\right) & \left(1-t^{2}+3t,1-t^{2}+3t\right) & \left(t^{2}-6t+5,t^{2}+4t\right)\\ \left(3-3t,2t+3\right) & \left(3t,5-2t\right) & \left(t^{2}+4t,t^{2}-6t+5\right) & \left(3-t^{2}-t,3-t^{2}-t\right) \end{array}\right). \end{array}$$

*There is exactly one pure NE at a pair of strategies (3,3). Here again, the maximum payoff is the same for both players, is reached at t=1/2, and is equal to $2\frac{1}{4}$. Table 8 illustrates three strategy profiles in $D_{1}$, $E_{1}$, and $E_{2}$ class extensions for which NE are unique and feasible, along with the corresponding values of the parameter t that result in maximum equal payoffs for the players.*

## 5. Conclusions

Quantum game theory exhibits a high level of complexity because it integrates multiple scientific domains including physics, computer science, mathematics, and economics. This interdisciplinary nature creates a significant barrier to entry for researchers interested in exploring this field. However, alongside the advancements within the realm of emerging technologies, especially quantum computing, there is a growing emphasis on comprehending their associated threats and benefits. Potential users most commonly perceive the strategic implications of quantum computing in terms of its impact on security. In addition, anticipated gains exist concerning the acceleration of calculations. Globally, various research institutions provide strategic plans that detail milestones for information security teams in preparation for impending quantum threats. Conversely, quantum key distribution offers exceptionally secure key distribution and has been evaluated through pilot projects to serve as a foundation for encrypting user data.

In a context where quantum developments are primarily seen as threats rather than new opportunities, we put forward a theory of quantum games. This approach suggests that by integrating classical and quantum strategies, players can access a range of novel possibilities for achieving their objectives. The primary objective of quantum game theory application is to enhance individual payoff, adhering to a Nash equilibrium strategy, while simultaneously improving social welfare in accordance with a Pareto-optimal solution.

Our research aimed to explore quantum extensions of the standard format of the Prisoner’s Dilemma game through the integration of two unitary strategies into its classical version [28]. We determined all possible combinations of quantum strategies that lead to Nash equilibria in pure strategies. These equilibria are observed in all possible extension categories, with the exception of the $D_{2}$ class. The prerequisites for the existence of the previously mentioned equilibria are generally intricate, involving several interactions between the payoffs of the traditional game and specific parameters ($\theta_{i}$ and $\alpha_{i}$) associated with the unitary strategies. Additionally, we examined the significance of equal payoffs in Nash equilibria for extended versions of the standard Prisoner’s Dilemma, as specified by Equation (16). Our findings suggest that these payoffs attain a maximum value of 5/2, thereby exhibiting a closer alignment with Pareto-optimal solutions than the conventional Nash equilibrium outcome of the Prisoner’s Dilemma, which stands at 1. Nonetheless, achieving Pareto-optimal values, specifically 3 in this context, remains unattainable.

These findings can serve as a foundational basis for further exploration of NE, which can also be expressed using mixed strategies. A compelling research direction would be to determine if such NE can be more closely aligned with Pareto-optimal solutions compared to the results derived from employing pure strategies.

## Figures and Tables

**Table 1:** Summary of the conditions, for which the given strategy profiles in $A_{1}$ and $A_{2}$ class extensions are NE. For the existence of equilibria, the conjunction of the conditions given in columns p and r (PD payoffs (15)) and *a* (parameter (33) defining unitary strategies (3)) must be satisfied.

Strategy Profile	*p*	*r*	*a*
$A_{1}(2,2)\;$ $A_{2}\left(2,2\right)$	(0,r)	$\left(\frac{1}{2},1\right)$	{1}
$A_{1}(2,3)\;$ $A_{1}\left(3,2\right)$ $A_{2}(2,4)\;$ $A_{2}\left(4,2\right)$	$\left(0,\frac{1}{6}\right]$	$\left(\frac{1}{2},1-3p\right]$	$\left[\frac{1}{4},\frac{1-r}{1+p-r}\right]$
		(1−3p,1−p]	$\left[\frac{p}{1+p-r},\frac{1-r}{1+p-r}\right]$
	$\left(\frac{1}{6},\frac{1}{2}\right)$	$\left(\frac{1}{2},1-p\right]$	$\left[\frac{p}{1+p-r},\frac{1-r}{1+p-r}\right]$
$A_{1}(2,4)\;$ $A_{1}\left(4,2\right)$ $A_{2}(2,3)\;$ $A_{2}\left(3,2\right)$	(0,r)	$\left(\frac{1}{2},\frac{3-p}{3}\right)$	$\left\{1\right\}$
		$\left\{\frac{3-p}{3}\right\}$	$\left\{\frac{1}{4},1\right\}$
		$\left(\frac{3-p}{3},1\right)$	$\left[\frac{1-r}{1+p-r},1\right]$
$A_{1}\left(3,3\right)$ $A_{2}\left(4,4\right)$	$\left[0,\frac{1}{6}\right]$	[1−3p,1)	$\left[\frac{1}{2}-\frac{1}{2}\sqrt{\frac{p}{1+p-r}},\frac{1}{4}\right]$
	$\left[\frac{1}{6},\frac{1}{2}\right]$	$\left(\frac{1}{2},1\right)$	
	$\left(\frac{1}{2},r\right)$	$\left(p,1\right)$	
$A_{1}(3,4)\;$$A_{1}(4,3)\;$ $A_{2}\left(4,3\right)$ $A_{2}\left(3,4\right)$	$\left(0,\frac{1}{6}\right)$	$\left(\frac{1}{2},1-3p\right]$	$\left\{\frac{1}{4}\right\}$
$A_{1}\left(4,4\right)$ $A_{2}\left(3,3\right)$	(0,r)	$\left(\frac{1}{2},\frac{3}{4}\right]$	$\left[\frac{1}{2}+\sqrt{\frac{1-r}{1+p-r}},1\right]$
	(0,3−3r)	$\left(\frac{3}{4},1\right)$	
	{3−3p}	$\left(\frac{3}{4},1\right)$	$\left[\frac{1}{2}+\sqrt{\frac{1-r}{1+p-r}},1\right]\cup \left\{\frac{1}{4}\right\}$
	(3−3r,r)		$\left[\frac{1}{4},\frac{1}{2}-\sqrt{\frac{1-r}{1+p-r}}\right]\cup \left[\frac{1}{2}+\sqrt{\frac{1-r}{1+p-r}},1\right]$

**Table 2:** NE with maximal and equal payoffs and the corresponding *a* parameters for the $A_{1}$ class extension of the PD (16); the symbol ✗ denotes lack of an NE for the corresponding strategy profiles.

✗	✗	✗	✗
**✗**	(1,1) for a=1	$\left(\frac{5}{2},\frac{5}{2}\right)$ for $a=\frac{1}{2}$	(1,1) for a=1
**✗**	$\left(\frac{5}{2},\frac{5}{2}\right)$ for $a=\frac{1}{2}$	$\left(\frac{5}{3},\frac{5}{3}\right)$ for $a=\frac{3-\sqrt{3}}{6}$	**✗**
**✗**	(1,1) for a=1	**✗**	$\left(\frac{5}{3},\frac{5}{3}\right)$ for $a=\frac{3+\sqrt{6}}{6}$

**Table 3:** The *B* class strategy parameters resulting in NE. Parameters *p* and *r* are PD payoffs, the mark **✗** denotes the lack of an NE for the corresponding pair of strategies and the mark **✓** denotes that NE exists for all parameter values.

✗	✗	✗	✗
**✗**	$p\geq \frac{1+r}{3}$	$p\leq \frac{1+r}{3}$	$p\leq \frac{1+r}{3}$
**✗**	$p\leq \frac{1+r}{3}$	**✓**	**✓**
**✗**	$p\leq \frac{1+r}{3}$	**✓**	**✓**

**Table 4:** NE payoffs in the class *B* extension for the standard PD (16). The mark **✗** denotes the lack of an NE for the corresponding pair of strategies.

✗	✗	✗	✗
**✗**	**✗**	$\left(2\frac{1}{4},2\frac{1}{4}\right)$	$\left(2\frac{1}{4},2\frac{1}{4}\right)$
**✗**	$\left(2\frac{1}{4},2\frac{1}{4}\right)$	$\left(2\frac{1}{4},2\frac{1}{4}\right)$	$\left(2\frac{1}{4},2\frac{1}{4}\right)$
**✗**	$\left(2\frac{1}{4},2\frac{1}{4}\right)$	$\left(2\frac{1}{4},2\frac{1}{4}\right)$	$\left(2\frac{1}{4},2\frac{1}{4}\right)$

**Table 5:** The *C* class strategy parameters resulting in NE. Parameters *p* and *r* are directly related to PD payoffs (15), while *t* refers to EWL scheme parameter $\theta_{1}$ (23). The mark **✗** denotes the lack of an NE for the corresponding strategy profile.

✗	✗	✗	✗
**✗**	$\left(p>\frac{1}{2}\right.$∧$\left.\frac{r-p}{p+r-1}\leq t\leq \frac{2p-1}{p+r-1}\right)$ ∨ $\left(p=\frac{r+1}{3}\right.$ ∧ $\left.t=\frac{1}{2}\right)$	$\left(0<p\leq 1-r\wedge t\geq \frac{1}{2}\right)$∨ $\left(1-r<p\leq \frac{r+1}{3}\wedge t=\frac{1}{2}\right)$	$\left(0<p\leq 1-r\wedge t\leq \frac{1}{2}\right)$∨ $\left(1-r<p\leq \frac{r+1}{3}\wedge t=\frac{1}{2}\right)$
**✗**	$\left(0<p\leq 1-r\wedge t\geq \frac{1}{2}\right)$∨ $\left(1-r<p\leq \frac{r+1}{3}\wedge t=\frac{1}{2}\right)$	0<p<r<1 ∧2r>1 $\;\wedge \;t=\frac{1}{2}$	0<p<r<1 ∧2r>1 $\;\wedge \;t=\frac{1}{2}$
**✗**	$\left(0<p\leq 1-r\wedge t\leq \frac{1}{2}\right)$∨ $\left(1-r<p\leq \frac{r+1}{3}\wedge t=\frac{1}{2}\right)$	0<p<r<1 ∧2r>1 $\;\wedge \;t=\frac{1}{2}$	0<p<r<1 ∧2r>1 $\;\wedge \;t=\frac{1}{2}$

**Table 6:** NE payoffs in the class *C* extension for the standard PD (16). The mark **✗** denotes the lack of an NE for the corresponding pair of strategies.

✗	✗	✗	✗
**✗**	**✗**	$\left(2\frac{1}{2},2\frac{1}{2}\right)$	$\left(2\frac{1}{4},2\frac{1}{4}\right)$
**✗**	$\left(2\frac{1}{2},2\frac{1}{2}\right)$	$\left(2\frac{1}{4},2\frac{1}{4}\right)$	$\left(2\frac{1}{4},2\frac{1}{4}\right)$
**✗**	$\left(2\frac{1}{4},2\frac{1}{4}\right)$	$\left(2\frac{1}{4},2\frac{1}{4}\right)$	$\left(2\frac{1}{4},2\frac{1}{4}\right)$

**Table 7:** Summary of the criteria for which the specified strategy profiles in the *D* and *E* class extensions constitute NE. The presence of equilibria requires that the conditions outlined in columns *p* and *r* (PD payoffs (15)) along with the parameter *t* are fulfilled.

Strategy Profile	*p*	*r*	*t*
$D_{1}\left(2,2\right)$	(0,r)	(p,1)	(0,1)
$D_{2}$	–	–	–
$E_{1}\left(4,4\right)$	(0,r)	(p,1)	$\left[\frac{1}{2},1\right)$
$E_{2}\left(3,3\right)$	(0,r)	(p,1)	$\left(0,\frac{1}{2}\right]$

**Table 8:** NE with maximal and equal payoffs and the corresponding *t* parameters for the $D_{1}$, $E_{1}$, and $E_{2}$ class extensions of the PD (16). The symbol **✗** denotes the lack of an NE for the corresponding pair of strategies.

✗	✗	✗	✗
**✗**	(1,1) for $t=1\;(D_{1})$	**✗**	**✗**
**✗**	**✗**	$\left(2\frac{1}{4},2\frac{1}{4}\right)$ for $t=\frac{1}{2}\;\left(E_{2}\right)$	**✗**
**✗**	**✗**	**✗**	$\left(2\frac{1}{4},2\frac{1}{4}\right)$ for $t=\frac{1}{2},\;\left(E_{1}\right)$

## Data Availability

Data is contained within the article.

## Appendix A. Proofs of the Propositions for the Existence of NE in Class *A*

### Appendix A.1. Proposition 3

**Proof.** Let 0≤a≤1, 0<p<r, and 0.5<r<1. Note that

(A1) ar−ap+p<1foreverya∈[0,1].

Moreover,

(A2) 1−a<ap−ar+rfora=1.

Equations (A1) and (A2) lead to conclusion that none of the strategies in the first row and column can be NE. □

### Appendix A.2. Proposition 4

**Proof.** Consider the following set of inequalities:

(A3) $$\begin{array}{cc} \; & p\geq 0, \end{array}$$

(A4) $$\begin{array}{cc} \; & p\geq 1-a, \end{array}$$

(A5) $$\begin{array}{cc} \; & p\geq ap-ar+r. \end{array}$$

Inequality (A5) can be rearranged to the following form:

(A6) (p−r)(1−a)≥0,

where it is evident that the inequality is fulfilled only for a=1. Inequalities (A3) and (A4) are also satisfied by a=1, which proves that the strategy profile (2,2) is an NE. □

### Appendix A.3. Proposition 5

**Proof.** Consider the following set of inequalities:

(A7) $$\begin{array}{cc} \; & a\geq (1-a)p+ar, \end{array}$$

(A8) $$\begin{array}{cc} \; & a\geq 4\left(a-a^{2}\right)p+{\left(1-2a\right)}^{2}r, \end{array}$$

(A9) $$\begin{array}{cc} \; & a\geq {\left(1-2a\right)}^{2}, \end{array}$$

(A10) $$\begin{array}{cc} \; & 1-a\geq (1-a)r+ap. \end{array}$$

Inequality (A9) is fulfilled by $a\in \left[\frac{1}{4},1\right]$. It is easy to show that ${\left(1-2a\right)}^{2}\geq 4\left(a-a^{2}\right)p+{\left(1-2a\right)}^{2}r$ if a∈[0,1] and $\left(1-a\right)p+ar\geq 4\left(a-a^{2}\right)p+{\left(1-2a\right)}^{2}r$ if $a\in \left[\frac{1}{4},1\right]$. Thus the above set of inequalities comes down to

(A11) $$\begin{array}{cc} \; & a\geq (1-a)p+ar, \end{array}$$

(A12) $$\begin{array}{cc} \; & \frac{1}{4}\leq a\leq 1, \end{array}$$

(A13) $$\begin{array}{cc} \; & 1-a\geq (1-a)r+ap. \end{array}$$

Inequalities (A11) and (A13) are satisfied for $\frac{p}{1+p-r}\leq a\leq 1$ and $0\leq a\leq \frac{r-1}{r-1-p}$, respectively. Note that $\frac{p}{1+p-r}\leq \frac{r-1}{r-1-p}$, if r≤1−p. Moreover, $\frac{p}{1+p-r}>\frac{1}{4}$, if r>1−3p and $0<p\leq \frac{1}{6}$. Thus, if $0<p\leq \frac{1}{6}$ and 1−3p<r≤1−p, then $\frac{p}{1+p-r}\leq \frac{r-1}{r-1-p}$. If $0<p\leq \frac{1}{6}$ and $\frac{1}{2}<r\leq 1-3p$, then $\frac{1}{4}\leq a\leq \frac{r-1}{r-1-p}$. If $\frac{1}{6}<p<\frac{1}{2}$ and $\frac{1}{2}<r<1-p$, and then $\frac{p}{1+p-r}\leq \frac{r-1}{r-1-p}$. □

### Appendix A.4. Proposition 6

**Proof.** Consider the following set of inequalities:

(A14) $$\begin{array}{cc} \; & ap-ar+r\geq (1-a), \end{array}$$

(A15) $$\begin{array}{cc} \; & ap-ar+r\geq 4(1-a)a, \end{array}$$

(A16) $$\begin{array}{cc} \; & ap-ar+r\geq {\left(1-2a\right)}^{2}p-4\left(a-1\right)ar, \end{array}$$

(A17) $$\begin{array}{cc} \; & ap-ar+r\geq p. \end{array}$$

Inequality (A17) is satisfied by a=1. Inequality (A16) or equivalently (r−p)(a−1)(4a−1)≥0 is satisfied by $a\in \left[0,\frac{1}{4}\right]\cup \left\{1\right\}$. Note that if $a\leq \frac{1}{4}$, inequality (A15) is also fulfilled. The solution of inequality (A14) is $a\in \left[\frac{1-r}{1+p-r},1\right]$. Moreover, notice that $\frac{1-r}{1+p-r}<\frac{1}{4}$, if $r>\frac{3-p}{3}$. Finally, pairs of strategies (2,4) and (4,2) are NE if

(A18) $$\begin{array}{c} \left\{\begin{array}{cc} a=1 & \mathrm{for}\;0<r<\frac{3-p}{3},\\ a=1\vee a=\frac{1}{4} & \mathrm{for}\;r=\frac{3-p}{3},\\ a\in \left[\frac{1-r}{1+p-r},1\right] & \mathrm{for}\;\frac{3-p}{3}<r<1. \end{array}\right. \end{array}$$

□

### Appendix A.5. Proposition 7

**Proof.** Consider the following set of inequalities:

(A19) $$\begin{array}{cc} \; & r-4a(a-1)(p-r)\geq ar-ap+p, \end{array}$$

(A20) $$\begin{array}{cc} \; & r-4a(a-1)(p-r)\geq a, \end{array}$$

(A21) $$\begin{array}{cc} \; & r-4a\left(a-1\right)\left(p-r\right)\geq {\left(1-2a\right)}^{2}. \end{array}$$

Inequality (A19) can be transformed into (r−p)(a−1)(4a−1)≥0, which is satisfied by $a\in \left[0,\frac{1}{4}\right]\cup \left\{1\right\}$. Notice that ${\left(1-2a\right)}^{2}\geq a$ if $a\leq \frac{1}{4}$. Thus, pair of strategies (3,3) is NE if inequalities (A19) and (A21) are satisfied. Inequality (A21) is equivalent to $4a^{2}\left(r-p-1\right)-4a\left(r-p-1\right)+r-1\geq 0$ and its solution is the interval $\left[\frac{1}{2}-\frac{1}{2}\sqrt{\frac{p}{1+p-r}},\frac{1}{2}+\frac{1}{2}\sqrt{\frac{p}{1+p-r}}\right]$. Note that $\frac{1}{2}-\frac{1}{2}\sqrt{\frac{p}{1+p-r}}<\frac{1}{4}$, if r>1−3p. Finally, a pair of strategies (3,3) is an NE if the following conditions are satisfied:

(A22) $$\begin{array}{c} \left\{\begin{array}{cc} a=\frac{1}{4} & \mathrm{for}\;\;0<p<\frac{1}{6}\wedge r=1-3p,\\ \frac{1}{2}-\frac{1}{2}\sqrt{\frac{p}{1+p-r}}\leq a\leq \frac{1}{4} & \mathrm{for}\;\;0<p<\frac{1}{6}\wedge 1-3p<r<1,\\ \frac{1}{2}-\frac{1}{2}\sqrt{\frac{p}{1+p-r}}\leq a\leq \frac{1}{4} & \mathrm{for}\;\;\frac{1}{6}\leq p\leq \frac{1}{2}\wedge \frac{1}{2}<r<1,\\ \frac{1}{2}-\frac{1}{2}\sqrt{\frac{p}{1+p-r}}\leq a\leq \frac{1}{4} & \mathrm{for}\;\;\frac{1}{2}<p<r\wedge p<r<1. \end{array}\right. \end{array}$$

□

### Appendix A.6. Proposition 8

**Proof.** Consider the following set of inequalities:

(A23) $$\begin{array}{cc} \; & 4(1-a)a\geq 1-a, \end{array}$$

(A24) $$\begin{array}{cc} \; & 4(1-a)a\geq ap-ar+r, \end{array}$$

(A25) $$\begin{array}{cc} \; & 4\left(1-a\right)a\geq {\left(1-2a\right)}^{2}p-4\left(a-1\right)ar, \end{array}$$

(A26) $$\begin{array}{cc} \; & {\left(1-2a\right)}^{2}\geq ar-ap+p, \end{array}$$

(A27) $$\begin{array}{cc} \; & {\left(1-2a\right)}^{2}\geq a, \end{array}$$

(A28) $$\begin{array}{cc} \; & {\left(1-2a\right)}^{2}\geq r-4\left(a-1\right)a\left(p-r\right). \end{array}$$

Inequality (A23) can be written as $-4a^{2}+5a-1\geq 0$, and so, $a\in \left[\frac{1}{4},1\right]$. Inequality (A27) is equivalent to $4a^{2}-5a+1\geq 0$, which is satisfied by $a\in \left[0,\frac{1}{4}\right]$. Moreover, (A25) is not satisfied if a=1. Thus, $a=\frac{1}{4}$. Finally, setting $a=\frac{1}{4}$ for the remaining inequalities leads to the conclusion that a pair of strategies (3,3) is an NE, if $a=\frac{1}{4}$, $\frac{1}{2}<r\leq 1-3p$, $0<p<\frac{1}{6}$. □

### Appendix A.7. Proposition 9

**Proof.** Consider the following set of inequalities:

(A29) $$\begin{array}{cc} \; & {\left(1-2a\right)}^{2}p-4\left(a-1\right)ar\geq ap-ar+r, \end{array}$$

(A30) $$\begin{array}{cc} \; & {\left(1-2a\right)}^{2}p-4\left(a-1\right)ar\geq 1-a, \end{array}$$

(A31) $$\begin{array}{cc} \; & {\left(1-2a\right)}^{2}p-4\left(a-1\right)ar\geq 4\left(1-a\right)a. \end{array}$$

Inequality (A29) can be transformed to

(A32) $$\left(p-r\right)\left(4a^{2}-5a+1\right)\geq 0$$

and its solution is $\left[\frac{1}{4},1\right]$. Notice that if $a\geq \frac{1}{4}$, then 4a(1−a)≥1−a. Thus, a pair of strategies (4,4) is an NE if inequalities (A29) and (A31) are satisfied. Inequality (A31) is equivalent to

(A33) $$4a^{2}\left(p-r+1\right)-4a\left(p-r+1\right)+p\geq 0,$$

of which the solution is $\left[\frac{1}{2}-\frac{1}{2}\sqrt{\frac{1-r}{p-r+1}},\frac{1}{2}+\frac{1}{2}\sqrt{\frac{1-r}{p-r+1}}\right]$. Note that

(A34) $$\begin{array}{cc} \frac{1}{2}+\frac{1}{2}\sqrt{\frac{1-r}{p-r+1}}\leq 1 & \;\mathrm{for}\;\;0<p<r, \end{array}$$

(A35) $$\begin{array}{cc} \frac{1}{2}-\frac{1}{2}\sqrt{\frac{1-r}{p-r+1}}>\frac{1}{4} & \;\mathrm{for}\;\;p>3-3r, \end{array}$$

(A36) $$\begin{array}{cc} \frac{1}{2}-\frac{1}{2}\sqrt{\frac{1-r}{p-r+1}}<\frac{1}{4} & \;\mathrm{for}\;\;p<3-3r, \end{array}$$

(A37) $$\begin{array}{cc} \frac{1}{2}-\frac{1}{2}\sqrt{\frac{1-r}{p-r+1}}=\frac{1}{4} & \;\mathrm{for}\;\;p=3-3r. \end{array}$$

Finally, a pair of strategies (4,4) is an NE if

(A38) $$\begin{array}{c} \left\{\begin{array}{cc} \frac{1}{2}+\frac{1}{2}\sqrt{\frac{1-r}{p-r+1}}\leq a\leq 1 & \mathrm{for}\;\;\frac{1}{2}<r\leq \frac{3}{4}\wedge 0<p<r,\\ \frac{1}{2}+\frac{1}{2}\sqrt{\frac{1-r}{p-r+1}}\leq a\leq 1 & \mathrm{for}\;\;\frac{3}{4}<r<1\wedge 0<p<3-3r,\\ a=\frac{1}{4}\vee \frac{1}{2}+\frac{1}{2}\sqrt{\frac{1-r}{p-r+1}}\leq a\leq 1 & \mathrm{for}\;\;\frac{3}{4}<r<1\wedge p=3-3r,\\ \frac{1}{4}\leq a\leq \frac{1}{2}-\frac{1}{2}\sqrt{\frac{1-r}{p-r+1}}\vee \frac{1}{2}+\frac{1}{2}\sqrt{\frac{1-r}{p-r+1}}\leq a\leq 1 & \mathrm{for}\;\;\frac{3}{4}<r<1\wedge 3-3r<p<r. \end{array}\right. \end{array}$$

□

## Appendix B. Proofs of the Propositions for the Existence of NE in Class *B*

### Appendix B.1. Proposition 10

**Proof.** The proof is based on the definition of NE and the inequalities that occur for PD defining parameters *r* and *p*.

i. The (2,1) profile is the unique NE candidate in the first column, as player 1 has the highest payoff of 1>r and $1>\frac{1+r+p}{4}$ in that column. Nevertheless, player 2’s payoff for this profile is 0—i.e., it is smaller than the payoffs of the other players in this row. Consequently, neither the (2,1) profile nor any other profile in the first column can be classified as NE. The symmetry of the game implies that no profile in the first row can also be classified as NE.
ii. The (2,2) is an NE if $p\geq \frac{1+r+p}{4}$, which leads directly to $p\geq \frac{1+r}{3}$
iii. The (3,2) is an NE if $\frac{1+r+p}{4}\geq p$ which leads directly to $p\leq \frac{1+r}{3}$. The remaining profiles are also NE, based on the same inequality.
iv. For these profiles the payoffs of both players in each row and column are identical, thereby demonstrating that these are NE.

□

## Appendix C. Proofs of the Propositions for the Existence of NE in Class *C*

### Appendix C.1. Proposition 11

**Proof.** Note that none of the pairs of strategies represented in the first row or the first column of the *C* class can be an NE because

(A39) $$\begin{array}{cc} \; & \frac{1}{2}\left(1-t\right)\left(p+r\right)+\frac{t}{2}<1, \end{array}$$

(A40) $$\begin{array}{cc} \; & \frac{t}{2}\left(p+r\right)+\frac{1-t}{2}t<1 \end{array}$$

for any t∈(0,1). Indeed, after some transformations, inequality (A39) takes on the form of (p+r−1)t−(p+r−2)>0. Since

(A41) $$\begin{array}{c} (p+r-1)t-(p+r-2)>(p+r-2)t-(p+r-2)=(p+r-2)(t-1)>0, \end{array}$$

inequality (A39) remains true for t∈(0,1). Simultaneously, inequality (A40) is equivalent to (p+r−1)t−1<0. Since (p+r−1)t−1<t−1, the solution of (A40) is given by t∈(0,1). □

### Appendix C.2. Proposition 12

**Proof.** Consider the following inequality:

(A42) $$p\geq \frac{1}{2}\left(1-t\right)\left(p+r\right)+\frac{t}{2},$$

or, equivalently, (p+r−1)t+p−r≥0. Let $p>\frac{1}{2}$, then $\frac{r-p}{p+r-1}\leq t<1$. Inequality $p\geq \frac{t}{2}\left(p+r\right)+\frac{1-t}{2}$ or its equivalent form (p+r−1)t+1−2p≤0 is fulfilled if $0<t\leq \frac{2p-1}{p+r-1}$ and $p>\frac{1}{2}$. Consequently, a pair of strategies given by the element (2,2) of *C* class is an NE when $\frac{r-p}{p+r-1}\leq t\leq \frac{2p-1}{p+r-1}$ with the assumption of $p>\frac{1+r}{3}$. Furthermore, if $p=\frac{1+r}{3}$, then the pair of strategies given by the element (2,2) results in an NE if $t=\frac{1}{2}$. □

### Appendix C.3. Proposition 13

**Proof.** In what follows, we will prove that the intersection of the set of inequalities

(A43) $$\begin{array}{cc} \; & pt^{2}+r{\left(1-t\right)}^{2}+t\left(1-t\right)\geq \frac{t}{2}\left(p+r\right)+\frac{1-t}{2}, \end{array}$$

(A44) $$\begin{array}{cc} \; & pt^{2}+r{\left(1-t\right)}^{2}+t\left(1-t\right)\geq \frac{1}{2}\left(1-t\right)\left(p+r\right)+\frac{t}{2}, \end{array}$$

(A45) $$\begin{array}{cc} \; & pt^{2}+r{\left(1-t\right)}^{2}+t\left(1-t\right)\geq t\left(1-t\right)\left(p+r\right)+{\left(1-t\right)}^{2}, \end{array}$$

is given by $t=\frac{1}{2}$. Inequality (A43) is equivalent to (2t−1)(t(p+r−1)−2r+1)≥0. Since

(A46) $$\begin{array}{c} (p+r-1)t-(2r-1)<(2r-1)t-(2r-1)<(2r-1)(t-1)<0, \end{array}$$

and if t∈(0,1), then 2t−1≤0, and hence, $t\in \left(0,\frac{1}{2}\right]$. Inequality (A45) is equivalent to (2t−1)(t(p+r−1)−(r−1))≥0. Since

(A47) $$\begin{array}{c} (p+r-1)t-(r-1)>(r-1)t-(r-1)=(r-1)(t-1)>0, \end{array}$$

this inequality holds for every $t\in \left[\frac{1}{2},1\right)$. It is easy to check that the remaining inequality (A44) is fulfilled for $t=\frac{1}{2}$. Hence, we can conclude that a pair of strategies (3,3) results in an NE if $t=\frac{1}{2}$. □

### Appendix C.4. Proposition 14

**Proof.** In what follows, we will prove that the intersection of the set of inequalities

(A48) $$\begin{array}{cc} \; & p{\left(1-t\right)}^{2}+rt^{2}+t\left(1-t\right)\geq \frac{1}{2}\left(1-t\right)\left(p+r\right)+\frac{t}{2} \end{array}$$

(A49) $$\begin{array}{cc} \; & p{\left(1-t\right)}^{2}+rt^{2}+t\left(1-t\right)\geq \frac{t}{2}\left(p+r\right)+\frac{1-t}{2} \end{array}$$

(A50) $$\begin{array}{cc} \; & p{\left(1-t\right)}^{2}+rt^{2}+t\left(1-t\right)\geq \left(1-t\right)t\left(p+r\right)+t^{2} \end{array}$$

is given by $t=\frac{1}{2}$. Inequality (A48) can be rewritten in the form of (2t−1)(t(p+r−1)+r−p)≥0. One can note that

(A51) $$\begin{array}{cc} & t(p+r-1)+r-p=tp+tr-t+r-p=p(t-1)+2tr-tr-t+r\\ & =p(t-1)-r(t-1)+t(2r-1)=(p-r)(t-1)+t(2r-1)>0. \end{array}$$

Hence, one can conclude that inequality (A48) is fulfilled for $t\in \left[\frac{1}{2},1\right)$. Inequality (A50) is equivalent to (2t−1)(t(p+r−1)−p)≥0. Note that

(A52) p+r−1<p,foreveryt∈(0,1).

Consequently, we can conclude that the solution of (A50) is given by the following intersection $\left(0,\frac{1}{2}\right]$. One can easily check that the remaining inequality (A49) is true for $t=\frac{1}{2}$. Hence, we conclude that the pair of strategies given by the element (4,4) is an NE if $t=\frac{1}{2}$. □

### Appendix C.5. Proposition 15

**Proof.** Consider the following set of inequalities:

(A53) $$\begin{array}{cc} \; & \frac{1}{2}\left(1-t\right)\left(p+r\right)+\frac{t}{2}\geq \frac{t}{2}\left(p+r\right)+\frac{1-t}{2} \end{array}$$

(A54) $$\begin{array}{cc} \; & \frac{1}{2}\left(1-t\right)\left(p+r\right)+\frac{t}{2}\geq pt^{2}+r{\left(1-t\right)}^{2}+t\left(1-t\right) \end{array}$$

(A55) $$\begin{array}{cc} \; & \frac{1}{2}\left(1-t\right)\left(p+r\right)+\frac{t}{2}\geq t\left(1-t\right)\left(p+r\right)+{\left(1-t\right)}^{2} \end{array}$$

(A56) $$\begin{array}{cc} \; & \frac{1}{2}\left(1-t\right)\left(p+r\right)+\frac{t}{2}\geq p \end{array}$$

Inequality (A55) or equivalently (2t−1)(t(p+r−1)−(p+r−2))≥0 is satisfied for $t\in \left[\frac{1}{2},1\right)$. Let now $\frac{1}{2}\leq t<1$. If r<1−p, then inequality (A53), equivalent to (2t−1)((p+r−1)t+p−r)≤0, is satisfied. Inequality (A54) is equivalent to (2t−1)((p+r−1)t+p−r)≤0, which is satisfied for $\frac{1}{2}\leq t<1$ and r<1−p. Moreover, inequality (A56), with the equivalent form of (p+r−1)t+p−r≤0, is also satisfied for $\frac{1}{2}\leq t<1$ and r<1−p. Consequently, we infer that if 0<p<1−r and $\frac{1}{2}\leq 0<1$, then (3,2) and (2,3) are NE of the *C* game. It is easy to prove that if $t=\frac{1}{2}$ and $1-r<p<\frac{1+r}{3}$, then (3,2) and (2,3) are NE of the *C* game. □

### Appendix C.6. Proposition 16

**Proof.** Consider the following set of inequalities:

(A57) $$\begin{array}{cc} \; & \frac{t}{2}\left(p+r\right)+\frac{1-t}{2}\geq \frac{1}{2}\left(1-t\right)\left(p+r\right)+\frac{t}{2}, \end{array}$$

(A58) $$\begin{array}{cc} \; & \frac{t}{2}\left(p+r\right)+\frac{1-t}{2}\geq \left(1-t\right)t\left(p+r\right)+t^{2}, \end{array}$$

(A59) $$\begin{array}{cc} \; & \frac{t}{2}\left(p+r\right)+\frac{1-t}{2}\geq p{\left(1-t\right)}^{2}+rt^{2}+t\left(1-t\right) \end{array}$$

(A60) $$\begin{array}{cc} \; & \frac{t}{2}\left(p+r\right)+\frac{1-t}{2}\geq p,\; \end{array}$$

One notes that inequality (A58) is equivalent to (2t−1)((p+r−1)t−1≥0 and is satisfied for $t\in \left(0,\frac{1}{2}\right]$. Let $0<t\leq \frac{1}{2}$ and r<1−p. Then, inequality (A57), equivalent to (2t−1)(p+r−1)≥0, is satisfied. Since t(p+r−1)+1−2p>t(2p−1)−(2p−1)=(2p−1)(t−1) and 2p−1≤1−2r<0, inequality (A60) is true for $t\in \left(0,\frac{1}{2}\right]$. From this, we immediately get the solution of equation (1−2t)(t(p+r−1)−(2p−1))≥0, equivalent to (A59), namely, $\left(0,\frac{1}{2}\right]$. It is easy to see that if $t=\frac{1}{2}$ and $1-r<p<\frac{1+r}{3}$, then (4,2) and (2,4) are NE of the *C* game. □

### Appendix C.7. Proposition 17

**Proof.** Below it will be proved that the following set of inequalities is satisfied for $t=\frac{1}{2}$.

(A61) $$\begin{array}{cc} \; & \left(1-t\right)t\left(p+r\right)+t^{2}\geq \frac{1}{2}\left(1-t\right)\left(p+r\right)+\frac{t}{2} \end{array}$$

(A62) $$\begin{array}{cc} \; & \left(1-t\right)t\left(p+r\right)+t^{2}\geq \frac{t}{2}\left(p+r\right)+\frac{1-t}{2} \end{array}$$

(A63) $$\begin{array}{cc} \; & \left(1-t\right)t\left(p+r\right)+t^{2}\geq p{\left(1-t\right)}^{2}+rt^{2}+t\left(1-t\right) \end{array}$$

(A64) $$\begin{array}{cc} \; & t\left(1-t\right)\left(p+r\right)+{\left(1-t\right)}^{2}\geq \frac{t}{2}\left(p+r\right)+\frac{1-t}{2} \end{array}$$

(A65) $$\begin{array}{cc} \; & t\left(1-t\right)\left(p+r\right)+{\left(1-t\right)}^{2}\geq \frac{1}{2}\left(1-t\right)\left(p+r\right)+\frac{t}{2} \end{array}$$

(A66) $$\begin{array}{cc} \; & t\left(1-t\right)\left(p+r\right)+{\left(1-t\right)}^{2}\geq pt^{2}+r{\left(1-t\right)}^{2}+t\left(1-t\right) \end{array}$$

Inequality (A61) is equivalent to (2t−1)((p+r)−t(p+r−1))≥0. One notes that t(p+r−1)−(p+r)<0; therefore 2t−1≥0, and hence $t\in \left[\frac{1}{2},1\right)$. Inequality (A65) can be rewritten in the following form: (2t−1)((p+r−1)t−(p+r−2))≤0. Since (p+r−1)t−(p+r−2)>0, inequality (A65) is satisfied if $t\in (0,\frac{1}{2}]$. It can be easily proved that the remaining inequalities are satisfied for $t=\frac{1}{2}$. Hence, one can infer that pairs of strategies (3,4) and (4,3) are NE for $t=\frac{1}{2}$. □

## Appendix D. Proofs of the Propositions for the Existence of NE in Class *D*

### Appendix D.1. Proposition 18

**Proof.** It is easily seen that

(A67) $$\begin{array}{cc} \; & p>p-pt, \end{array}$$

(A68) $$\begin{array}{cc} \; & p>pt \end{array}$$

for every t∈(0,1). Therefore, a profile strategy (2,2) is an NE for every t∈(0,1). Since the inequalities (r−1)t+1<1, (1−r)t<1 are satisfied for t∈(0,1), we conclude that neither (1,j) nor (i,1), i,j∈{1,2,3,4}, is an NE. We aim to prove the following inequalities:

(A69) $$\begin{array}{cc} \; & rt<(1-p)t+p, \end{array}$$

(A70) $$\begin{array}{cc} \; & \left(r-1\right)t^{2}+t+p{\left(1-t\right)}^{2}<\left(1-p\right)t+p, \end{array}$$

(A71) $$\begin{array}{cc} \; & \left(1-p-r\right)t^{2}+\left(p+r\right)t<\left(1-p\right)t+p \end{array}$$

for every t∈(0,1). The inequality (A69) is equivalent to (r+p−1)t<p. From (A52) we have

(A72) (r+p−1)t−p<pt−p=p(t−1)<0.

Hence, (A69) holds for every t∈(0,1). Inequality (A70) is equivalent to t((r+p−1)t−p)<0. From (A72) we conclude that (A70) holds for every t∈(0,1). Inequality (A71) is equivalent to

(A73) $$-\left(t-1\right)\left((p+r-1)t-p\right)<0.$$

From (A72) it follows that 0<t<1. From (A67) and (A69)–(A71) we conclude that neither (3,j) nor (i,3), i,j∈{1,2,3,4}, is an NE. Now we show that

(A74) $$\left(p-1\right)t^{2}+t+r{\left(1-t\right)}^{2}<\left(p-1\right)t+1$$

or, equivalently,

(A75) $$\left(t-1\right)\left((p+r-1)t-(r-1)\right)<0$$

holds for every t∈(0,1). From (A47), inequality (A74) holds for every t∈(0,1), and the strategy profile (4,4) is not an NE. □

### Appendix D.2. Proposition 19

**Proof.** Since pt≤1 and p−pt≤1 hold for every t∈(0,1), it follows that neither (1,j) nor (i,1), i,j∈{1,2,3,4}, is an NE. The inequality

(A76) rt<(1−p)t+p

is equivalent to $-\left((p+r-1)t-p\right)>0$. We conclude from (A72) that the interval (0,1) is solution of this inequality, and finally neither (4,2) nor (2,4) is an NE. It is easily to seen that

(A77) (r−1)t−(p−1)>(p−1)t−(p−1)=(p−1)(t−1)>0

for every t∈(0,1). Hence a strategy profile (2,2) is not an NE. Consider

(A78) r−rt<(p−1)t+1

or equivalently, (p+r−1)t+(1−r)>0. From (A47) it follows that t∈(0,1); hence neither (3,2) nor (2,3) is an NE. We conclude from (A74) and (p−1)t+1<(r−1)t+1 for every t∈(0,1) that the strategy profile (4,4) is not an NE. The strategy profile (3,3) is not an NE, which follows from inequalities

(A79) $$\begin{array}{cc} \; & \left(r-1\right)t^{2}+t+p{\left(1-t\right)}^{2}\geq \left(1-p-r\right)t^{2}+\left(p+r\right)t\;\;\mathrm{for}\;\mathrm{every}\;\;t\in \left(0,\frac{1}{2}\right], \end{array}$$

(A80) $$\begin{array}{cc} \; & \left(r-1\right)t^{2}+t+p{\left(1-t\right)}^{2}\leq \left(p-1\right)t+1\;\;\mathrm{for}\;\mathrm{every}\;\;t\in \left(0,\frac{1}{2}\right]. \end{array}$$

It easily seen that the solution of (A79), or equivalently $\left(t-\frac{1}{2}\right)\left((r+p-1)t-p\right)<0$, is $\left(0,\frac{1}{2}\right]$. Note that the solution of (1−p)t+p≤(p−1)t+1 is $\left(0,\frac{1}{2}\right]$. We conclude from this and from (A71) that (A80) holds for every $t\in \left(0,\frac{1}{2}\right]$. Since the inequalities

(A81) $$\begin{array}{cc} \; & \left(1-p-r\right)t^{2}+\left(p+r\right)t\geq \left(r-1\right)t^{2}+t+p{\left(1-t\right)}^{2} \end{array}$$

(A82) $$\begin{array}{cc} \; & \left(p+r\right)\left(t-t^{2}\right)+{\left(1-t\right)}^{2}\leq \left(p-1\right)t^{2}+t+r{\left(1-t\right)}^{2} \end{array}$$

hold for every $t\in \left[\frac{1}{2},1\right)$, it follows that neither (4,3) nor (3,4) is an NE. Consider (A81), or equivalently, $\left(-1+2t\right)\left((p+r-1)t-p\right)\leq 0$. From (A72) it follows that $t\in \left[\frac{1}{2},1\right)$. Moreover, from (A47) we conclude that (A82), or equivalently $\left(2t-1\right)\left((p+r-1)t+1-r\right)\geq 0$, holds for every $t\in \left[\frac{1}{2},1\right)$. □

## Appendix E. Proofs of the Propositions for the Existence of NE in Class *E*

### Appendix E.1. Proposition 20

**Proof.** Let us first prove that the following inequalities hold:

(A83) $$\begin{array}{cc} \; & \left(p-1\right)t^{2}+t+r{\left(1-t\right)}^{2}\geq p-pt\;\;\mathrm{for}\;\mathrm{every}\;\;\frac{1}{2}\leq t<1, \end{array}$$

(A84) $$\begin{array}{cc} \; & \left(p-1\right)t^{2}+t+r{\left(1-t\right)}^{2}\geq pt\;\;\mathrm{for}\;\mathrm{every}\;\;0\leq t<1, \end{array}$$

(A85) $$\begin{array}{cc} \; & \left(p-1\right)t^{2}+t+r{\left(1-t\right)}^{2}\geq \left(p+r\right)\left(t-t^{2}\right)+{\left(1-t\right)}^{2}\;\;\mathrm{for}\;\mathrm{every}\;\;\frac{1}{2}\leq t<1. \end{array}$$

Inequality (A84) is equivalent to $\left(t-1\right)\left((p+r-1)t-r\right)\geq 0$. Clearly

(A86) (p+r−1)t−r<rt−r=r(t−1)<0,

and hence (A84) holds for every t∈(0,1). From (A47) it follows that (A85), or equivalently

(A87) $$\left(1-2t\right)\left((p+r-1)t+r-1\right)\geq 0$$

, holds for every $t\in \left[\frac{1}{2},1\right)$. Since p(1−t)≤pt for every $t\in \left[\frac{1}{2},1\right)$, the solution of (A83) is $\left[\frac{1}{2},1\right)$. From (A83)–(A85) we conclude that the strategy profile (4,4) is an NE for every $t\in \left[\frac{1}{2},1\right)$. It is clear that the following inequalities hold for every t∈(0,1):

(A88) $$\begin{array}{cc} \; & (1-p)t+p<1,\\ \; & rt<1 \end{array}$$

Hence neither (1,j) nor (i,1), i,j∈{1,2,3,4}, is an NE. From (A84) we see that strategy profiles (4,2) and (2,4) are not NE. From (A47) it follows that

(A89) (p+r−1)t−r+1≥0

holds for every t∈(0,1). Hence strategy profiles (3,2) and (2,3) are not NE. From (A81) and (A82) we conclude that neither (3,4) nor (4,3) is an NE. Since

(A90) $$\left(r-1\right)t^{2}+t+p{\left(1-t\right)}^{2}\leq \left(r-1\right)t+1$$

for every t∈(0,1), it follows that the strategy profile (3,3) is not an NE. Indeed, (A90) is equivalent to $\left(t-1\right)\left((p+r-1)t+1-p\right)\leq 0.$ It follows easily that

(A91) (p+r−1)t+1−p>(p−1)t−(p−1)=(p−1)(t−1)>0,

and hence t∈(0,1). □

### Appendix E.2. Proposition 21

**Proof.** Let us first prove that the following inequalities hold:

(A92) $$\begin{array}{cc} \; & \left(r-1\right)t^{2}+t+p{\left(1-t\right)}^{2}\geq p-pt\;\;\mathrm{for}\;\mathrm{every}\;\;0<t<1, \end{array}$$

(A93) $$\begin{array}{cc} \; & \left(r-1\right)t^{2}+t+p{\left(1-t\right)}^{2}\geq pt\;\;\mathrm{for}\;\mathrm{every}\;\;0<t\leq \frac{1}{2}, \end{array}$$

(A94) $$\begin{array}{cc} \; & \left(r-1\right)t^{2}+t+p{\left(1-t\right)}^{2}\geq \left(1-p-r\right)t^{2}+\left(p+r\right)t\;\;\mathrm{for}\;\mathrm{every}\;\;0<t\leq \frac{1}{2}. \end{array}$$

Inequality (A92) is equivalent to $t\left((p+r-1)t+1-p\right)\geq 0.$ Since p+r−1>r−1 and p−1<r−1,

(A95) (p+r−1)t−(p−1)>(p−1)t−(p−1)=(p−1)(t−1)>0.

Hence t∈(0,1). Consider (A94), or equivalently $\left(2t-1\right)\left((p+r-1)t-p\right)\geq 0$. From (A52) we have

(A96) (p+r−1)t−p<pt−p=p(t−1)<0.

It follows that $t\in \left(0,\frac{1}{2}\right]$. Note that pt≤p(1−t) where $t\in \left(0,\frac{1}{2}\right]$. Hence

(A97) $$\left(r-1\right)t^{2}+t+p{\left(1-t\right)}^{2}\geq p-pt\geq pt\;\;\mathrm{for}\;\mathrm{every}\;\;\frac{1}{2}\leq t<1.$$

From (A92)–(A94), we conclude that the strategy profile (3,3) is an NE for every $t\leq \frac{1}{2}$. It is easily seen that the following inequalities hold for every t∈(0,1):

(A98) $$\begin{array}{cc} \; & (p-1)t+1<1,\\ \; & r-rt<1. \end{array}$$

Hence neither (1,j) nor (i,1), i,j∈{1,2,3,4}, is an NE. Since p≤(1−p)t+p for every t∈(0,1), the strategy profile (2,2) is not an NE. From (A92) neither (3,2) nor (2,3) is an NE. Clearly, from (A96) it follows that (4,2) and (2,4) are not NE (A96). Moreover, from (A81) and (A82) we conclude that strategy profiles (4,3) and (3,4) are not NE. The following inequality holds for every t∈(0,1):

(A99) $$\left(p-1\right)t^{2}+t+r{\left(1-t\right)}^{2}\leq \left(r-1\right)t+1$$

Indeed, from (A74) and (p−1)t+1<(r−1)t+1, it follows that (A99) holds. Hence the strategy profile (4,4) is not an NE. □

## References

[1] Eisert J., Wilkens M., Lewenstein M. (1999). Quantum Games and Quantum Strategies. Phys. Rev. Lett..

[2] Flitney A.P., Abbott D. (2002). An introduction to quantum game theory. Fluct. Noise Lett..

[3] Meyer D.A. (1999). Quantum Strategies. Phys. Rev. Lett..

[4] Li H., Du J., Massar S. (2002). Continuous-variable quantum games. Phys. Lett. A.

[5] Frąckiewicz P., Rycerz K., Szopa M. (2021). Quantum absentminded driver problem revisited. Quantum Inf. Process..

[6] Fadaki M., Abbasi B., Chhetri P. (2022). Quantum game approach for capacity allocation decisions under strategic reasoning. Comput. Manag. Sci..

[7] Iqbal A., Toor A. (2001). Evolutionarily stable strategies in quantum games. Phys. Lett. A.

[8] Ikeda K., Aoki S. (2021). Infinitely repeated quantum games and strategic efficiency. Quantum Inf. Process..

[9] Chen L., Ang H., Kiang D., Kwek L., Lo C. (2003). Quantum prisoner dilemma under decoherence. Phys. Lett. A.

[10] Benjamin C., Dash A. (2020). Thermodynamic susceptibility as a measure of cooperative behavior in social dilemmas. Chaos Interdiscip. J. Nonlinear Sci..

[11] Altintas A.A., Ozaydin F., Bayindir C., Bayrakci V. (2022). Prisoners’ Dilemma in a Spatially Separated System Based on Spin–Photon Interactions. Photonics.

[12] Iqbal A., Chappell J.M., Abbott D. (2018). The equivalence of Bell’s inequality and the Nash inequality in a quantum game-theoretic setting. Phys. Lett. A.

[13] Chen H., Jia W. (2025). Dynamics of quantum Bertrand duopoly games with asymmetric information. Quantum Inf. Process..

[14] Xu J.M., Zhen Y.Z., Yang Y.X., Cheng Z.M., Ren Z.C., Chen K., Wang X.L., Wang H.T. (2022). Experimental Demonstration of Quantum Pseudotelepathy. Phys. Rev. Lett..

[15] Bugu S., Ozaydin F., Kodera T. (2020). Surpassing the classical limit in magic square game with distant quantum dots coupled to optical cavities. Sci. Rep..

[16] Makram-Allah A.T.M., Abd-Rabbou M.Y., Metwally N. (2024). Time dependence of Eisert–Wilkens–Lewenstein quantum game. Quantum Inf. Process..

[17] Tiago G., Naskar J., Maioli A.C., Balthazar W.F., Schmidt A.G.M., Huguenin J.A.O. (2025). Classical and quantum multiplayer Colonel Blotto game in all-optical setup. Quantum Inf. Process..

[18] Flitney A.P., Hollenberg L.C.L. (2007). Nash equilibria in quantum games with generalized two-parameter strategies. Phys. Lett. A.

[19] Frąckiewicz P. (2016). Remarks on quantum duopoly schemes. Quantum Inf. Process..

[20] Szopa M. (2021). Efficiency of Classical and Quantum Games Equilibria. Entropy.

[21] Landsburg S. (2011). Nash equilibria in quantum games. Proc. Am. Math. Soc..

[22] Bolonek-Lasoń K., Kosiński P. (2017). Mixed Nash equilibria in Eisert-Lewenstein-Wilkens (ELW) games. J. Phys. Conf. Ser..

[23] Nash J.F. (1950). Equilibrium points in n-person games. Proc. Natl. Acad. Sci. USA.

[24] Axelrod R., Dawkins R. (2006). The Evolution of Cooperation: Revised Edition.

[25] Li A., Yong X. (2015). Entanglement Guarantees Emergence of Cooperation in Quantum Prisoner’s Dilemma Games on Networks. Sci. Rep..

[26] Szopa M. (2014). How Quantum Prisoner’s Dilemma Can Support Negotiations. Optimum. Stud. Ekon..

[27] Frąckiewicz P., Szopa M. (2024). Permissible extensions of classical to quantum games combining three strategies. Quantum Inf. Process..

[28] Frąckiewicz P., Gorczyca-Goraj A., Szopa M. (2025). Permissible four-strategy quantum extensions of classical games. Quantum Inf. Process..

[29] Huang D.x., Tan B.q. (2024). “Fight alone” to “win–win cooperation”: A quantum stag hunt game model for analyzing cooperative R&D between enterprises. Quantum Inf. Process..

[30] Maschler M., Solan E., Zamir S. (2020). Game Theory.

[31] Van Damme E. (1987). Stability and Perfection of Nash Equilibria.

[32] Nash J. (1951). Non-Cooperative Games. Ann. Math..

[33] Osborne M.J., Rubinstein A. (1994). A Course in Game Theory.

[34] Binmore K., Binmore K. (2007). Playing for Real: A Text on Game Theory.

[35] Hardin G. (1968). The Tragedy of the Commons. Science.

